# Use of a Zwitterionic Isoquinolinium Dye as a Solvatochromic Indicator: Structure of the Microsphere of Solvation and Explicit Solvation Modeling

**DOI:** 10.1021/acs.jpcb.6c01103

**Published:** 2026-05-14

**Authors:** Ovidiu Gabriel Avadanei, Dana Ortansa Dorohoi, Mihaela Iuliana Avadanei

**Affiliations:** † Faculty of Physics, 112930 “Alexandru Ioan Cuza” University of Iasi, 11 Carol I Blvd, Iasi 700506, Romania; ‡ “Petru Poni” Institute of Macromolecular Chemistry, 41A Gr. Ghica Voda, Iasi 700487, Romania

## Abstract

The present study uses a hybrid approach to investigate the negative solvatochromism and preferential solvation of iso-quinolinium dicarboethoxy methylid (iQ) in pure and binary solvent mixtures. UV–vis absorbance studies were combined with DFT and TD-DFT methods with implicit and explicit solvation models. The binary solvent mixtures, formed from inert and protic polar solvent pairs, were treated within the Bosch–Rosés preferential solvation models. The solvatochromic analysis revealed that the behavior in binary mixtures is described by the general model, suggesting that the two solvents interact and the electrostatic forces are involved. Application of the statistical cell model confirms that the composition of the microsolvation sphere of iQ in binary mixtures consists mainly of the protic solvent. The DFT study in the implicit solvation model reproduced the excitation energies of iQ reasonably well. Explicit solvation studies using benzene and water revealed the stabilization of iQ in the inert solvent, the preferred interaction sites of water with iQ, and the effect of the location of explicit solvent molecules on the calculated absorption spectra.

## Graphical abstract

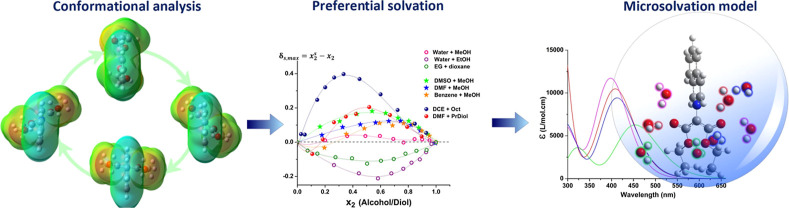

## 1 Introduction

Ylids with an (iso)­quinoline scaffold, a class of molecules with positive and negative charges adjacent to each other, are valuable building blocks in small organic and medicinal chemistry.^1–3^ A large subclass of isoquinolinium compounds are natural compounds, found in plant families such as barberry or poppy, from which morphine, codeine, or berberine can be obtained.^4^ In traditional medicine, these plants have been used for their analgesic, antimicrobial, anticancer, and anti-inflammatory properties. Zwitterionic stabilization of isoquinolinium molecules in solution is essential for therapeutic applications, and the drug effectiveness is attributed to the charge-separated structure.^5,6^ In the development of new isoquinolinium derivatives from natural and synthetic sources with pharmacological properties, the type of the charged units and the distance and chemical groups between them are essential for determining the physical properties.

Investigating intramolecular charge transfer in molecules where the donor and acceptor are separated by a single bond is particularly challenging. Many zwitterionic structures with short charge separations and a twisted ground-state geometry, such as quinolinium or pyridazinium ylid derivatives, exhibit pronounced negative solvatochromism and a complete lack of fluorescence.^7–10^ This last property prevents the use of some isoquinolinium derivatives as fluorescent sensors and fluorophores in chemical imaging. In contrast, their solvatochromism is a valuable tool for analyzing structure–property relationships and the solvation phenomena.

Following our interest in the photophysics of nonfluorescent compounds with charge-separated structures and their potential use as solvatochromic probes and sensors,^7–10^ we analyzed the solvents’ effects on an isoquinolinium ylid in binary and ternary systems. Quinolinium dicarboethoxy methylid (iQ), pictured in Chart 1a, is a zwitterionic compound with a very low presence in the literature but with a high scientific potential due to its particular structure. iQ exhibits an intramolecular charge transfer from the carbanion to the nitrogen atom of the polyheterocycle, as evidenced by a shift of the corresponding band to higher energy.^7,11^ The species sketched in Chart 1a coexists in fact with a series of resonance forms (Chart 1b), where the charges can be delocalized within the polyheterocylic system. The mesomeric forms are in a fast exchange with each other, and their population depends on the properties of the solvents in the microsphere of solvation. This large chemical space provides a playground for various solvation phenomena, which are primarily influenced by the hydrogen bond donor ability and the polarizability/dipolarity of the solvent. The carbanion site possesses the H-bond accepting property, so Platts and Howard^12^ and Rozas et al.^13^ have reported that stable H-bonded complexes were formed even with weak HB donor partners. The quaternized nitrogen atom is a strong electron acceptor and can interact with solvent molecules, thereby affecting the magnitude of the charge transfer within the iQ molecule.^14^ Papadakis has advanced several modes in which the positively charged quaternary nitrogen atom interacts with hydroxylic or amide-containing solvents.^14,15^ Still, reaching the N^+^ and C^–^ sites in iQ for H-bonding or electrostatic interactions is difficult due to the steric hindrance imposed by the dicarboethoxy arms. Second, the double ester moiety in the carboethoxy chains is a very good H-bond acceptor, with the Hunter electrostatic basicity parameter, β_2_^H^, of 0.45.^16^ On this ground, it is supposed that the interactions of iQ with a solvent with a significant basicity will deviate from those considered by Platts,^12^ Rozas et al.,^13^ and Papadakis,^14,15^ by primarily H-bonding at the ester site.

**Chart 1:**
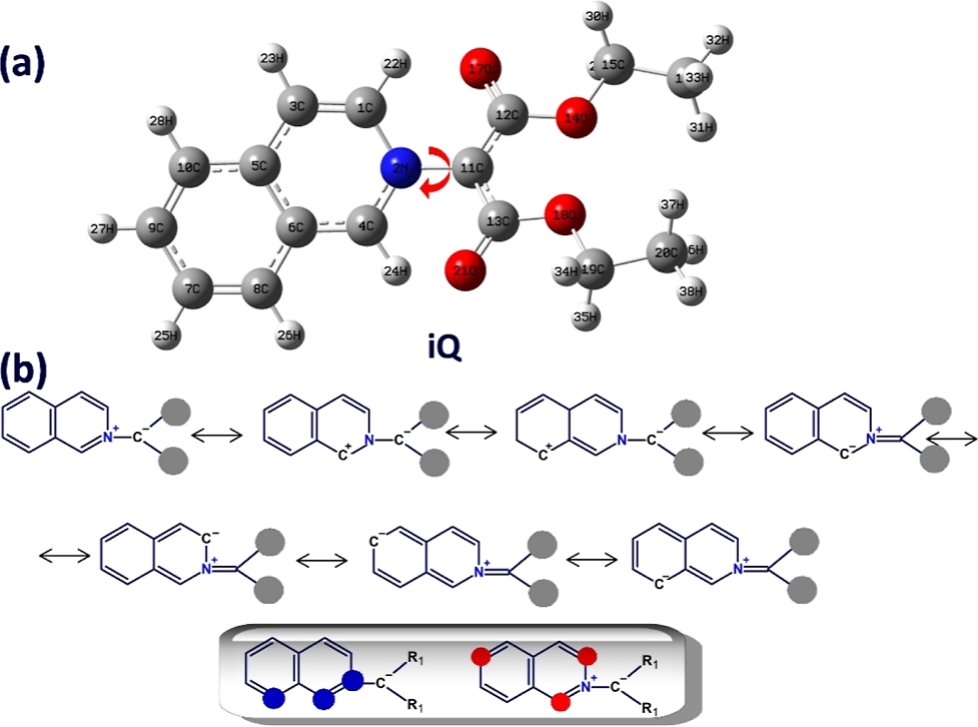
Chemical Structure of iQ (a) and the Relevant Resonance Structures, Showing the Possible Localization Sites of the Positive (Blue) and Negative Charges (Red) (Inset) (b); the Red Arrow Shows the Main Rotatable Bond (C^–^–N^+^)

The negative solvatochromic behavior of iQ in a large range of solvents was previously reported using the linear solvation energy relationship, and it was observed that the specific interactions are dominant in protic solvents.^7,11^ A preferential solvation by the polar protic solvent was also observed in several binary mixtures by using the statistical cell model of ternary solutions.^7,11^ Our understanding of how and to what extent zwitterions with charges separated by one single bond and concurrent H-bonding accepting groups in the side chains, such as iQ, interact with different kinds of solvents, and how charge transfer is modulated by these interactions, is still incomplete. The variation of the ionic charge induced by the solvent’s properties critically influences the uptake of quinolinium zwitterions when they are pharmacological agents or act as transporters of drugs and bioactive compounds.^6^ Knowledge of the solvation mechanism is crucial for designing dipolar bioactive quinolinium species with target properties. We decided to combine experimental studies with theoretical approaches to see how the solvent affects the iQ’s structure and electronic distribution, as well as the configuration of intermolecular hydrogen bonding. We have chosen eight binary mixtures, from which two are hydroalcoholic (water + methanol and water + ethanol) and the other six consist of apolar solvent and polar protic solvent pairs: benzene + methanol, 1,2-dichloroethane +1-octanol, and *N*,*N*-dimethylformamide + propane-1,3-diol. The solvatochromism in binary solution mixtures benzene + methanol, 1,2-dichloroethane +1-octanol, and *N*,*N*-dimethylformamide + propane-1,3-diol was studied using the statistical cell model of ternary solutions in previous reports.^7,11^

In this research, the composition of the microsolvation sphere of iQ across all ternary systems was analyzed for the first time using the modified Bosch–Rosés solvent exchange model.^17,18^ This method is especially suitable for solvent pairs that interact, such as alcohol–water mixtures, so that the intersolvent complex solvates the solute. Such a synergic behavior was observed in hydroalcoholic solutions of several negative solvatochromic dyes, such as 2,6-diphenyl-4-(2,4,6-triphenyl-1-pyridinio)­phenolate (Reichardt’s betaine dye), sodium {4-[4-(4-carboxylatophenyl)-2,6-diphenyl-1-pyridinio]-2,6-diphenylphenolate} (Reichardt’s water-soluble betaine dye), various nitrobenzene or aniline derivatives,^17,18^ or cyclodextrin-containing [2]­rotaxanes.^19^ Rosés et al. observed the enhancement of the water’s structure in the microsolvation sphere of these compounds, but this phenomenon is strong when the solute has poor H-bond acceptor properties. The zwitterionic ylid reported in our study has not a push–pull structure, such as Reichardt’s betaine dyes or aniline derivatives from the studies of Rosés et al.^17,18^ The short distance between the electron donor and acceptor groups and the presence of the carbonyl esters on the dicarboethoxy chains, which are H-bond acceptors, will influence the internal organization of hydroalcoholic mixtures in the first solvation shell, probably leading to preferential solvation.

To analyze the solute–solvent interactions at the molecular level and the electronic structure of iQ, we rely on quantum–mechanical calculations. Ab initio DFT methods with implicit solvation have been widely used, and several solvation models were developed for this purpose.^20^ Still, understanding the solvatochromism of solutes with reactive sites in coordinating solvents is not fully captured by implicit solvation alone as these methods were not specifically developed to account for intermolecular hydrogen bonding with the solvent. Implicit solvation speeds up molecular simulations but does not provide a deeper insight into showing the interacting functional groups. Second, TD/DFT calculations often show large deviations from experimental values of the energy and oscillator strength of electronic transitions due to the choice of functional and basis sets. Third, solute–solvent interactions may selectively occur at specific sites of the solute. As noted above, we reasoned that the substituent-selective solvation can modulate intramolecular charge transfer in iQ. To test this hypothesis, we used a combination of implicit and explicit solvation models. By placing actual solvent molecules around iQ, explicit solvation accounts for direct solute–solvent interactions, especially hydrogen bonding, but also for solvents that do not bind to iQ. The Minnesota charge-density-based implicit solvation model (SMD) of Marenich, Cramer, and Truhlar^21^ is suitable for solutions with nonelectrostatic interactions.^22^ We aimed to test the SMD approach for binding energy calculations using several solvation models in each case. We restricted the study to benzene and water, representing inert and strongly coordinating solvents, respectively. The number of solvent molecules in the microsolvation sphere was varied from two to eight. The hybrid approach employed in this study is computationally expensive, but it can elucidate the extent of intermolecular interactions and predict the excited states energies with reasonable accuracy.

The manuscript is organized as follows: (i) in the first part (Section 3.1), we conducted a conformational study of iQ to determine the most probable conformation in solution. The iQ molecule can be described as a molecular rotor based on the hypothesis that the two dicarboethoxy flaps substituted at the carbanion behave as a rigid fragment that can rotate and translate through coupled motion of the components.^23^ We examined the conformational changes in two different solvents: benzene and methanol. (ii) In the second part (Section 3.2), we analyze the solvatochromism in pure solvents by combining the experiments with DFT calculations. (iii) The third part (Section 3.3) is a solvatochromic study in binary solvent mixtures. (iv) In the fourth part (Section 3.4), we investigate the intermolecular interactions iQ –solvent using explicit solvation models in implicit solvation, in two homogeneous solutions.

We provide insights into the importance of explicit solute–solvent interactions in modeling the electronic structure of the solute and how the electric field experienced by the solute changes according to the solvent’s structure. There is a knowledge void regarding the properties and the solvation phenomena in the narrow class of isoquinolinium ylids with negative solvatochromism. The methodology used in this study allows for a more comprehensive understanding of how the structure of the solute molecule shapes the microsolvation sphere than using a single method alone.

## 2 Experimental Section

### 2.1 Materials

The synthesis and characterization of isoquinolinium dicarboethoxy methylid (iQ) have been previously reported.^7^ The organic solvents, of spectral grade, were acquired from Sigma-Aldrich, Alpha Aesar, and Merck and are as follows: 1,4-dioxane (Diox), benzene, 1,2-dichloroethane (DCE) (from Chemopar, Iasi, Romania), 1-octanol (Oct), ethanol (EtOH), methanol (MeOH), *N*,*N*-dimethylformamide (DMF), ethylene glycol (EG), propane-1,3-diol (PrDiol), dimethyl sulfoxide (DMSO), and Millipore double distilled water (produced in our laboratory).

### 2.2 Measurements

The concentration of iQ in pure solvents was maintained at 5 × 10^–5^ M, keeping the solution under the validity of the Lambert–Beer law. The UV–vis studies were conducted on an Analytic Jena Specord Plus-5 UV–vis spectrophotometer (Jena GmbH, Germany) using quartz cuvettes with a 10 mm path length. The binary mixtures were prepared by volume. The measurements were made at ambient temperature (22° ± 2 °C), at 0.2 nm resolution, with an uncertainty of ±0.5 nm. The reported values are averages of duplicate experiments.

### 2.3 Quantum Chemical Calculations

We used the Gaussian 09 Rev.A01 suite of programs.^24^ DFT geometry optimization of iQ in the gas phase and ten solvents, in the ground state, and calculation of single point energies were conducted applying TD-B3LYP^25^ and TD-CAM-B3LYP^26^ hybrid functionals with the Dunning’s aug-cc-pVDZ basis set.^27,28^ The energetic minimum was verified by calculating the harmonic frequencies and by the absence of an imaginary frequency. The vertical excitation energies have been calculated using TD-DFT in the adiabatic approximation for the first 15 states. To this end, we first conducted a comparative study using three hybrid functionals (CAM-B3LYP,^26^ PBEPBE,^29,30^ and WB97XD^31^) and the augmented correlation-consistent polarized valence double-ζ (aug-cc-pVDZ) basis set. CAM-B3LYP outperformed B3LYP and, to a greater extent, WB97XD, whereas PBEPBE gave results similar to those of B3LYP. The deviations in the calculated absorption maxima were approximately 40 nm using WB97XD and approximately 20 nm with PBEPBE. The results presented in the manuscript were obtained using the CAM-B3LYP hybrid functional.

Solvation effects were initially accounted for using the charge-density-based implicit solvation model (SMD), which was necessary to determine the Gibbs solvation energy at 298 K as the electronic energy difference between the solvent and gas phase (0 K).^21,22^ The intermolecular interactions between the solute molecule and the solvent were investigated using explicit solvation, with the counterpoise method and within the SMD model. The calculations were based on the TD-B3LYP-aug-cc-pVDZ-optimized geometries. The sensitivity of (TD)-DFT-CAM-B3LYP/aug-cc-pVTZ/SMD to the explicit solvent was tested by placing from two to eight solvent molecules in the vicinity of iQ, especially near the likeliest groups for interactions (carbonyl groups for HBD solvents) at the hydrogen bonding distance.

## 3 Results and Discussion

### 3.1 Conformational Analysis

The chemical structure of iQ, together with the atomic numbering, is given in Chart 1. The molecule consists of two important parts, the isoquinoline ring and the dicarbethoxy fragment. From a conformational point of view, the most relevant degree of freedom is the rotation of the dicarbethoxy segment about the C^–^–N^+^ bond, so iQ may have different configurations as a function of the orientation of N_1_–C_2_–C_11_–C_13_ and N_1_–C_2_–C_11_–C_12_ dihedral angles. To identify the most stable conformer of iQ, the rotational potential energy surface (rPES) was determined using the DFT/B3LYP/6-31G­(d,p)/SMD method in the gas phase and in two relevant solvents: benzene and methanol. The purpose was to determine how the type of solvent affects the rotational PES between the two parts of the molecule. First, the optimized ground-state geometry has an + *anti*-clinal (+*ac*) conformation, according to the Klyne–Prelog convention.^32^ The N_1_–C_2_–C_11_–C_13_ dihedral angle is 138.47°, the angle between the two carboethoxy flaps is 128°, and the length of the C^–^–N^+^ bond is 1.44(3) Å. The rotational potential energy surfaces of iQ with respect to ∠(N_1_–C_2_–C_11_–C_13_) in a complete rotation around the C^–^–N^+^ bond exhibit different profiles in the three cases analyzed. The maxima and minima are roughly in the same positions, but at different intensities (Figure 1a). For the dihedral angle varying from 0 to 180°, the rPES of the isolated molecule shows a central peak, with the highest energy barrier occurring at the perpendicular conformation (90°, 5.8 kcal/mol). The rPES of iQ in benzene is a rolling-hill type, with barriers to the flat (0°, syn, or 180°, anti) and perpendicular (90°) geometries of roughly the same value, around 3.16 kcal/mol. MeOH has a clear w-valley profile, with high energy barriers at the flat conformations (near 0°).^33^ The first conclusion is that the greatest rotational freedom of iQ occurs in benzene, and therefore in noncoordinating solvents, because the molecule can attain the syn/anti or perpendicular conformation by spending the same amount of energy.

**Figure 1:**
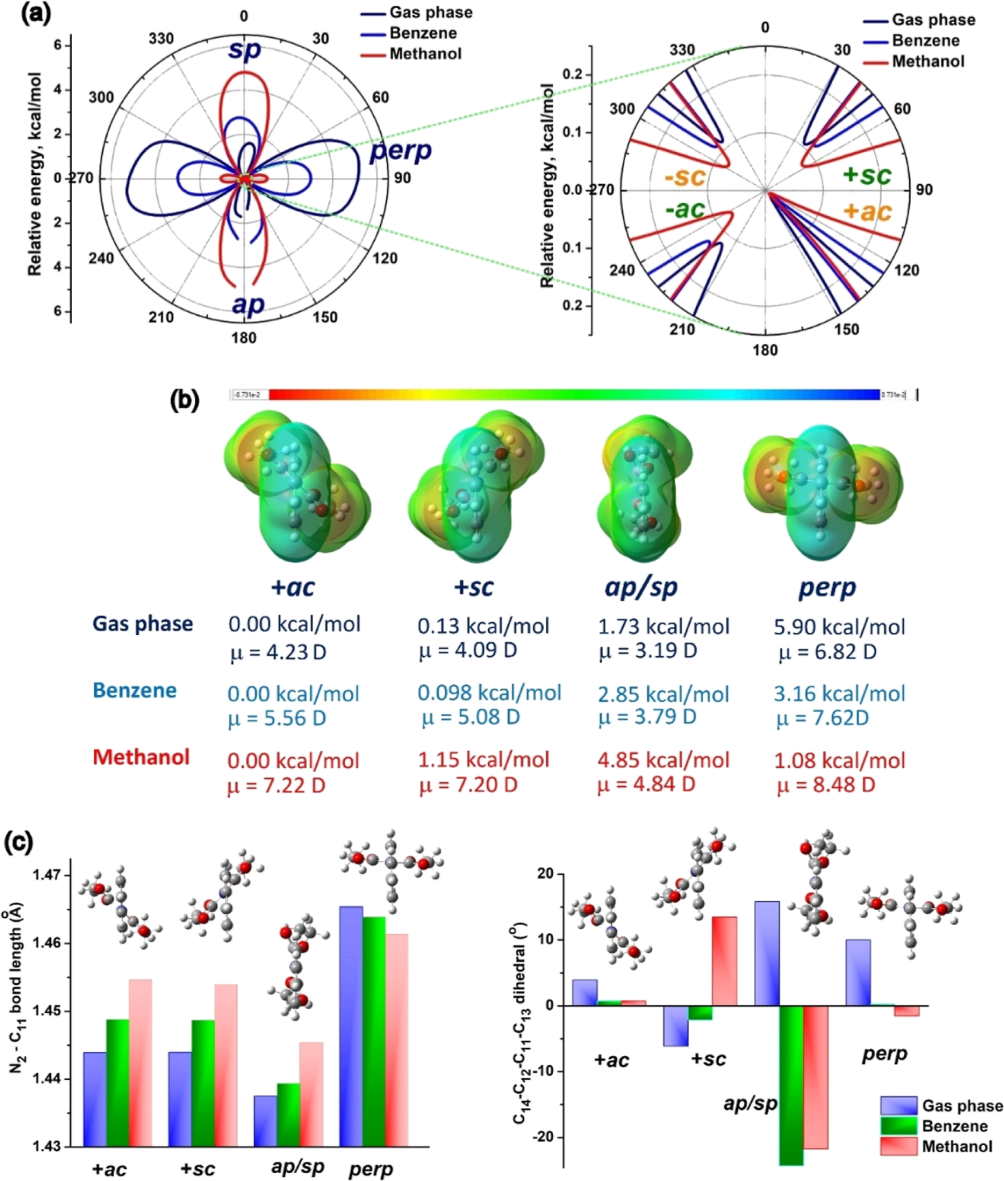
Rotational PES for iQ with respect to ∠(N_1_–C_2_–C_11_–C_13_) in the gas phase, benzene and methanol: (a) relative energy; (b) stable conformers with the lateral views of the optimized structures and the electrostatic potential surface maps, showing the electronic distribution, where μ is the dipole moment, in Debye; and (c) variation of the central C^–^–N^+^ bond (left) and ∠(C_14_–C_12_–C_11_–C_13_) dihedral angle of the dicarbethoxy part (right) as a function of conformation.

The rotational isomerism with respect to ∠(N_1_–C_2_–C_11_–C_13_) can form additionally three other stable conformers: – *anti*-clinal (−*ac*), + *syn*-clinal (+*sc*) ,and – *syn*-clinal (−*sc*). As ∠(N_1_–C_2_–C_11_–C_13_) is identical to ∠(N_1_–C_2_–C_11_–C_12_), the minima of the rPES are given by +*sc* and +*ac* conformations. The planar geometry, anti/syn-periplanar (sp) geometry, and orthogonal geometry (noted perp) are associated with high energy. Figure 1a,b shows that the rotational activation energy at ∠(N_1_–C_2_–C_11_–C_13_) = 0° is much higher in methanol (4.85 kcal/mol) than in benzene (2.85 kcal/mol). Conversely, the 90° barrier is high in the isolated state (5.90 kcal/mol) and decreases from 3.16 kcal/mol in benzene to 1.08 kcal/mol in methanol.

The stable +*sc* and +*ac* conformers are almost indistinguishable in relative energy and dipole moments; they differ in solvent-dependent rotational barriers and geometrical parameters. Figure 1c shows that the rotation of the dicarboethoxy fragment around the central C^–^–N^+^ bond induces variations of this bond’s length and of the dicarboethoxy part. The +*ac* to −*sc* transition requires overcoming a barrier of 5.79 kcal/mol in the gas phase, a 3.21 kcal/mol barrier in benzene, and a 1.07 kcal/mol barrier in MeOH, imposed by the perpendicular conformation. In this state, the central C^–^–N^+^ bond is longer than in +*sc* and +*ac*, minimizing the repulsion between the perpendicular fragments and increasing the electron delocalization. As shown by the electrostatic potential maps in Figure 1b, the net charge separation in perp provides the highest dipole moment among all geometries. The isolated molecule shows the highest barrier at 90°, reflecting difficulty in departing from the less twisted geometries, whereas the solvent molecules appear to facilitate rotation in solvated iQ. The high 0° barrier in methanol (4.85 kcal/mol) indicates that steric effects (repulsion) in the flat geometry are stronger than in benzene, most likely due to H-bonded solvent molecules to the carbonyl groups that stabilize the +*sc*/–*sc* and +*ac*/–*ac* conformers and create steric hindrance.

Furthermore, the geometry optimization of the energetic minima in the gas phase and ten solvents was performed within the DFT-B3LYP/6-311G­(d,p) framework. Due to the close values in the solvent’s parameters between ethylene glycol and propane-1,3-diol, we chose ethylene glycol as a representative for the two diols. The solvents’ parameters are listed in Table S1. The results of the geometry optimization are presented in Table 1. According to these data, the lowest energy of iQ is predicted in octanol, ethylene glycol, and DMF. Meanwhile, the dipole moment increases nonlinearly with solvent polarity, and the highest value, of 8.06 D, was observed in ethylene glycol.

**Table 1:** Calculated Dipole Moments, Relative Energies (*E* and Difference from the Gas Phase, Δ*E*), Relative Gibbs Energies (Δ*G*°), and Mulliken Charges of the N^+^ and C^–^ Sites of iQ; Model Chemistry: DFT­(B3LYP)/aug-cc-pVDZ

						Mulliken charge, *e*	
no.	solvent	dipole moment (D)	*E* (kcal/mol)	Δ*E* (kcal/mol)	Δ*G*° (kcal/mol)	N^+^	C^–^
0	gas	4.23	–612014.71	0	0	0.875	–0.687
1	1,4-dioxane	5.62	–612024.69	–9.98	–11.05	0.878	–0.692
2	benzene	5.56	–612019.99	–5.28	–5.09	0.873	–0.690
3	1,2-dichloroethane	6.82	–612025.65	–10.94	–11.62	0.870	–0.689
4	1-octanol	7.58	–612035.47	–20.76	–20.37	0.870	–0.688
5	ethanol	7.16	–612026.99	–12.28	–13.04	0.870	–0.689
6	methanol	7.22	–612027.22	–12.51	–13.25	0.870	–0.689
7	DMF	7.33	–612032.48	–17.77	–18.69	0.870	–0.685
8	ethylene glycol	8.06	–612033.06	–18.35	–17.85	0.863	–0.680
9	DMSO	7.27	–612027.46	–12.75	–13.11	0.868	–0.688
10	water	7.32	–612027.68	–12.97	–13.68	0.863	–0.685

### 3.2 Solvatochromism in Pure Solvents

The solvatochromism of iQ with respect to solvent polarity is evident from the experimental electronic absorption spectra in Figure 2a. The maximum of the ICT band (λ_max_) blue-shifts from 470 nm in benzene and 1,4-dioxane to around 414 nm in water and ethylene glycol. Dependence of λ_max_ with the dielectric permittivity of the solvent (Figure 2b) is generally linear but allows the formation of two plots with different slopes and of several clusters as a function of the H-bond donating ability of the solvent.

**Figure 2:**
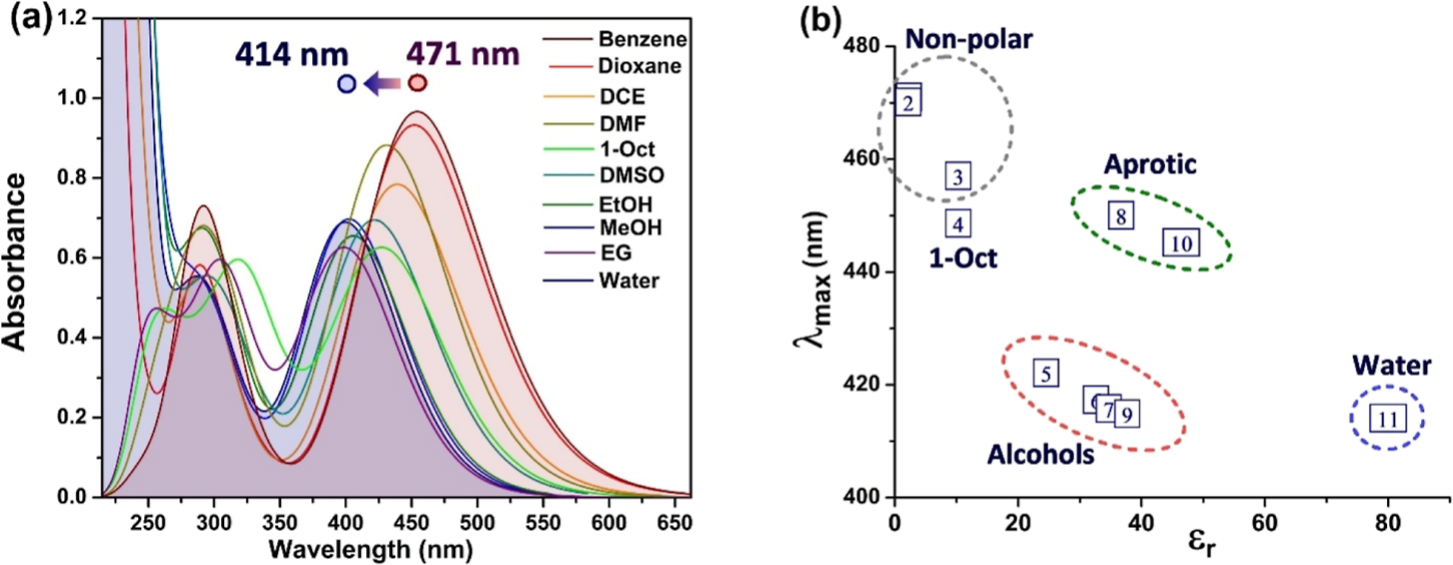
Experimental solvatochromism of iQ: (a) UV–vis absorption spectra; (b) plot of the maximum of the ICT band against the dielectric permittivity, ε_r_. The numbers correspond to the solvent’s enumeration position in Table S1.

The negative solvatochromism of iQ can be analyzed in relation to that of known solvatochromic dyes, such as Betaine 30. The molar transition energies (in kcal/mol) of the charge transfer band of iQ were calculated according to the formula *E*_T_ (kcal/mol) = 28590/λ_max_. The solvatochromic behavior of iQ shares no similarities with Betaine 30 because the relationship between *E*_T_ and the solvent polarity scale *E*_T_(30)^34–36^ (Figure S1) is not linear but follows a sigmoid function (*R* ^2^ = 0.980). For the linear part, obtained by excluding benzene, 1,4-dioxane, and water, the slope is *k* = 0.434, which is much less than that of Betaine 30 (*k* = 1.00),^34^ which suggests that the negative solvatochromism of iQ is weaker than that of Betaine 30. To analyze the types of interactions that contribute to the behavior of iQ, the Kamlet–Abboud–Taft multiparametric approach is written in the following expression:

1 $$E_{T}=E_{T}^{0}+a\cdot \pi^{*}+b\cdot \alpha +c\cdot \beta$$

where *E*_T_^0^ is the electronic transition energy in vacuum and α, β, and π* are the solvents’ properties: polarizability, H-bond donor (HBD), and H-bond acceptor (HBA) parameters, respectively. The *a*, *b*, and *c* factors are the regression parameters. Multiparametric fitting led to eq 2:

2 $$E_{T}=59.08+6.16\cdot \pi^{*}+7.19\cdot \alpha -1.75\cdot \beta$$

So, there is a hypsochromic shift induced by the medium polarity/polarizability and acidity, while the solvent’s basicity contributed to the bathochromic shift.

To analyze the electronic excitation leading to the ICT band, the electronic absorption spectra were calculated for the ten solvents under study using TD-DFT theory with the optimized ground-state geometry. Within the TD-DFT/CAM-B3LYP/aug-cc-pVDZ approach with SMD implicit solvation, the calculated spectra reproduce the experimental spectra reasonably well in terms of the relative intensity of the visible band but show small deviations in position (Table 2). The hypsochromic shift was reproduced by the SMD environment. Generally, the absorption maxima were overestimated in nonpolar solvents and were underestimated in protic solvents with Δλ_max_ = 15–25 nm. The lowest excited state, S_1_/ICT, has the HOMO–LUMO main configuration, with small contributions from LUMO+1 and LUMO+2 orbitals. The HOMO is an *n*-type orbital, with the electronic cloud distributed on the carbanion and less on the ester moieties. The LUMO is a π* orbital, being localized over the isoquinolinium ring (Figure 3).

**Table 2:** Calculated Absorption Data of iQ in the Solvents under Study in Comparison with the Gas Phase, Using TD-DFT/CAM-B3LYP/Aug-cc-pVDZ with the SMD Solvent Effect

solvent	λ_abs,exp_ (nm)	λ_abs,calc_ (nm)	oscillator strength, *f*	excitation energy (eV)	major MO configuration	*E*_HOMO_ (eV)	*E*_LUMO_ (eV)	Δ*E*_H–L_ (eV)
Gas		420	0.239	2.95	H → L (94.69%)	–6.49	–1.07	5.41
benzene	471	492	0.188	2.503	H → L (94.96%)	–7.57	–1.59	5.98
dioxane	470	496	0.130	2.495	H → L (93.03%)	–7.43	–1.46	5.97
DCE	457	440	0.150	2.819	H → L (93.72%)	–7.83	–1.41	6.41
1-octanol	448	416	0.149	2.404	H → L (94.37%)	–8.04	–1.23	6.80
ethanol	422	403	0.149	2.941	H → L (93.66%)	–7.89	–1.38	6.51
methanol	417	398	0.145	2.957	H → L (93.59%)	–7.90	–1.37	6.52
DMF	450	463	0.115	2.677	H → L (94.37%)	–7.59	–1.06	6.54
EG	415	391	0.346	3.169	H → L (88.65%)	–8.23	–1.18	7.05
DMSO	445	463	0.109	2.969	H → L (94.57%)	–7.91	–1.37	6.54
water	414	416	0.1498	2.979	H → L (93.62%)	–7.91	–1.36	6.55

**Figure 3:**
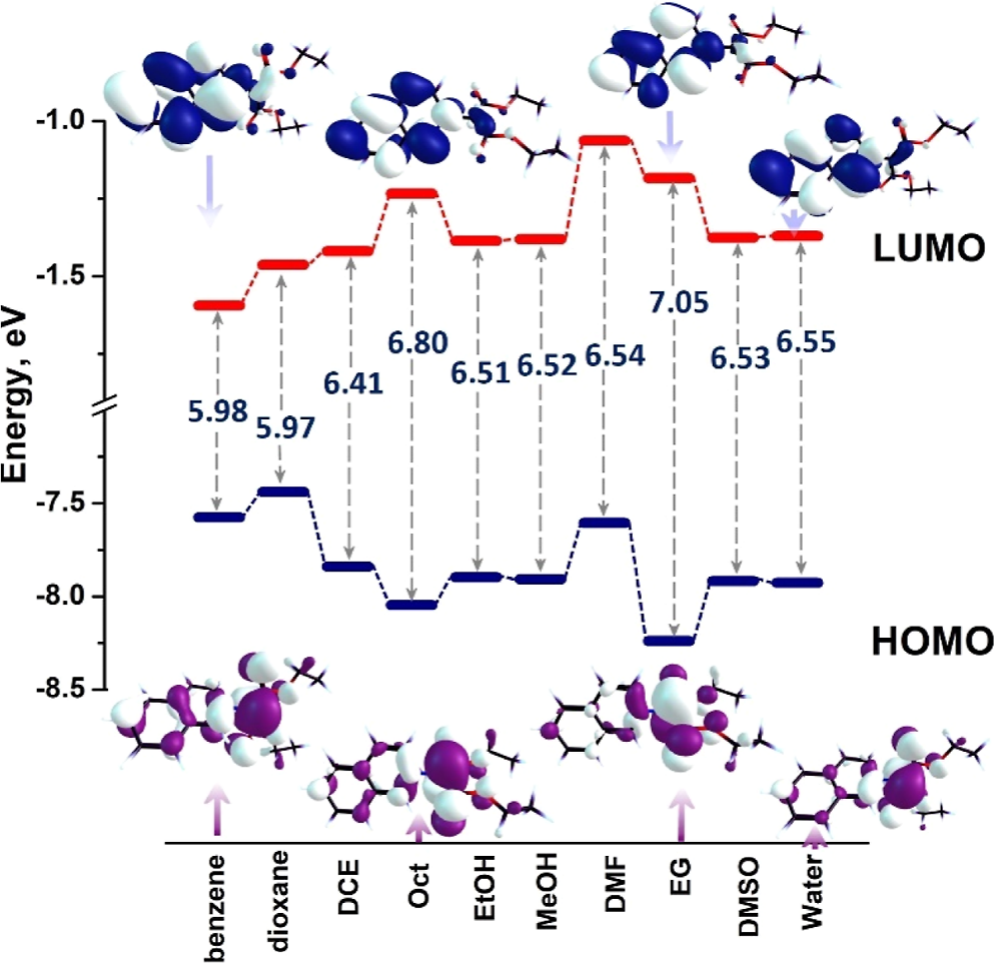
Energy of the frontier molecular orbitals of iQ involved in the iCT process in the individual solvents and pictures of the HOMO and LUMO orbitals in several relevant solvents. The energy of the HOMO–LUMO gap is in eV. Model chemistry: TD-DFT\CAM-B3LYP/aug-cc-pVDZ with SMD implicit solvation.

As shown in Figure 3, the S_1_ state arises from a charge-transfer process from the carbanion and ester site to the isoquinolinium ring. As solvent polarity increases, the HOMO and LUMO levels stabilize relative to benzene, and the greatest stabilization is observed in ethylene glycol. The separation between the HOMO and LUMO distributions reveals the charge transfer nature of the electronic excitation. The differences in the HOMO and LUMO levels, and their energy gap, are small among EtOH, MeOH, and water, suggesting similar excitation phenomena across the three solvents. In contrast, the HOMO–LUMO energy gap is the largest in 1-octanol and ethylene glycol, mainly due to the lowering of the HOMO. The negative solvatochromism is ascertained by the increase in the energy of the ground state and of the HOMO–LUMO gap in polar solvents.

Analysis of electronic excitation will be further developed using the correlated hole–electron pair^37,38^ as it provides a condensed perspective on the ICT electronic transition. The singular value decomposition of the one-particle transition density matrix gives the pairs of molecular orbitals that describe the one-electron transition: from the highest occupied natural transition orbital (HONTO) to the lowest unoccupied natural transition orbital (LUNTO). Consequently, the charge transfer matrix between the ground state and the first singlet excited state allows visualization of the electronic changes between the two states. Using Multiwfn 3.8 software,^39,40^ the wave functions of the ICT/S_1_ state for the hole–electron pairs from the transition density matrix were calculated for the solvents.

We have already seen that the S_0_–S_1_ transition is based on the HOMO to LUMO transition, but the hole and electron NTOs pictured in Figure S1 for the four representative solvents do not mirror the canonical orbitals. They are very similar across solvents, indicating that charge transfer is the main electronic event. The hole and electron densities are tightly localized on the C^–^–N^+^ bond and ester groups and the isoquinolinium ring, respectively. A closer look reveals differences in the HONTO distribution between benzene, a nonpolar solvent, and water or ethylene glycol, which are highly polar and have strong coordinating abilities. A large fraction of the hole is distributed across the polyheterocyclic system in polar solvents, lowering the charge transfer character of the S_0_ → S_1_ transition. Therefore, in coordinating solvents, the ICT/S_1_ state is a mix of charge transfer (major) and local excitation (minor). In conjunction with the distribution of holes and particles in each solvent, it is observed that the S_0_ → S_1_ transition has a pure charge transfer character in nonpolar and polar solvents with poor H-bonding abilities. As a consequence, the excited molecule becomes more polar than in the ground state, but the polarity is inverted compared with that in S_0_: the dipole moment is higher in nonpolar solvents than in polar solvents.

The heat map of the charge transfer matrix (CTM) for the six representative solvents, shown in Figure 4, illustrates the contributions of the iQ atoms to the charge-transfer process, excluding hydrogen atoms. For reference, the hole–electron densities with the atoms numbered are given above the CTM 2D-map. The CTMs are highly localized and lack diagonal elements, suggesting a pure S_0_ → S_1_ charge-transfer transition with minimal contamination from local excitation (Figure 4a–c).

**Figure 4:**
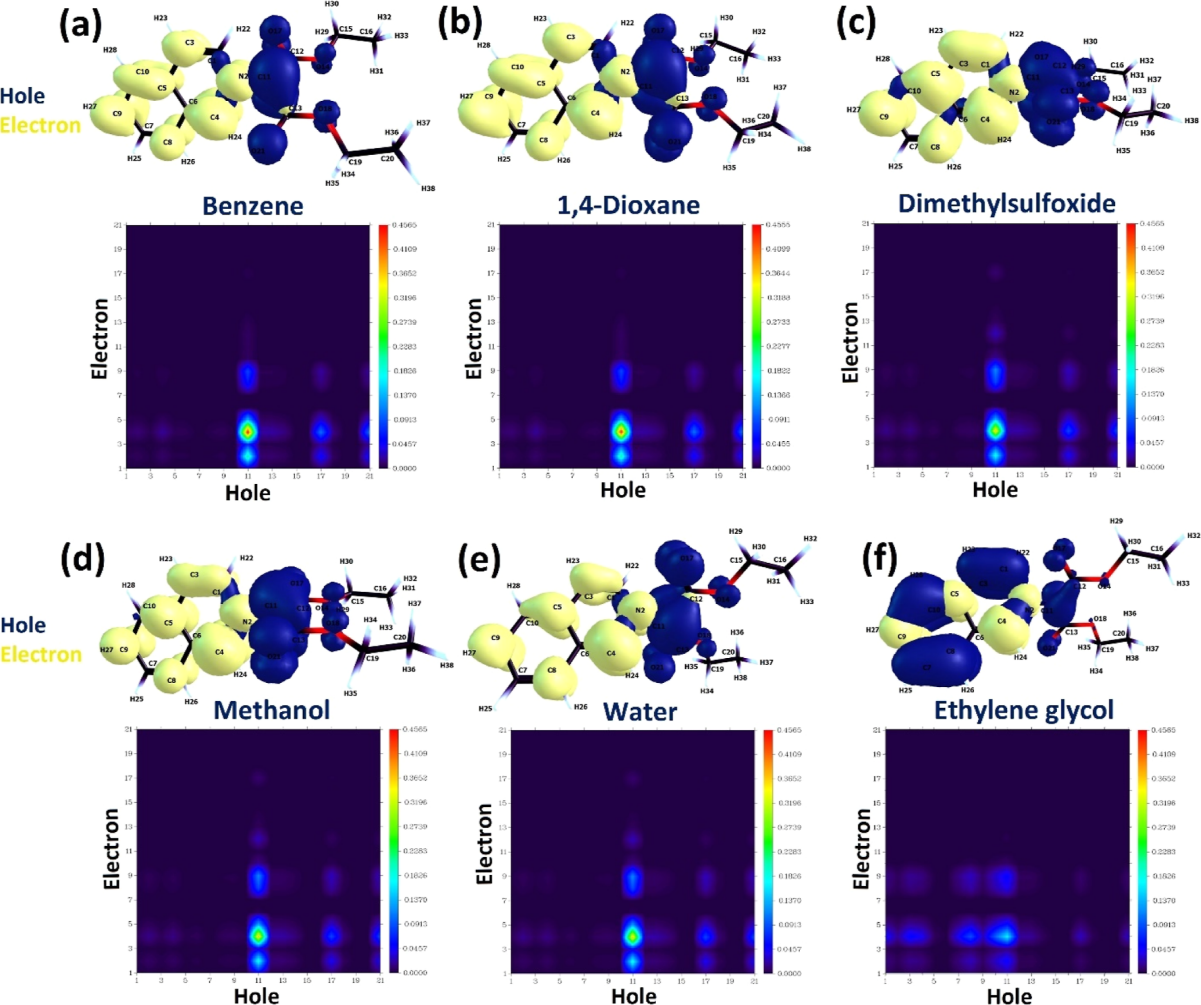
Heat maps representation of the charge transfer matrix of iQ in several solvents and the corresponding isosurface maps of the hole (navy) and electron (yellow) densities, with the atom numbering, in (a) benzene; (b) 1,4-dioxane; (c) dimethyl sulfoxide; (d) methanol; (e) water; and (f) ethylene glycol. The numbers in the abscissa and ordinate correspond to the indices of the C, N, and O atoms of iQ. The hydrogen atoms are excluded.

The CTM is localized mainly on atom no. 11, which is the carbanion, so the excited electrons are transferred to atoms with lower indices, from 1 to 9, which belong to the isoquinolinium ring. The pale spots on the electron side correspond to atoms no. 2 and 4, which are the N atom and C_4_ in the polyheterocycle, respectively, so the amount of transfer to this part is larger than in the rest of the system. This statement holds true for nonpolar solvents and agrees with the NTOs plot in Figure 4, but the CTM area on the carbanion faded in polar solvents (Figure 4d–f). As Figure 4 f shows, the hybridization of the excited state with local excitation occurred in ethylene glycol. A second bright spot appears on atom no. 17, which is the oxygen atom on the carboethoxy chain. It indicates that one of the two ester groups contributes to some extent to excitation into the S_1_ state, a contribution confirmed by the NTO plot in any solvent.

### 3.3 Electronic Excitation in Binary Solvent Mixtures

#### 3.3.1 Preferential Solvation Model

The quantum chemical calculations revealed that the charge-transfer process in iQ is strongly dependent on the solvent’s H-bonding abilities. The results are (i) small conformational changes induced by the direct interaction with the solvent; (ii) a contamination of the S_1_ state with local excitation in strong coordinating solvents, like alcohols, and, to a greater extent, in diols. The specific supramolecular interactions established by iQ will be analyzed in binary solvent mixtures, composed of a nonpolar solvent (S1) and a polar protic solvent (S2). The combinations were as follows: water + MeOH/EtOH; benzene/DMF/DMSO + MeOH; Diox + EG, DMF + PrDiol, and DCE + Oct. The results are shown in Tables S2–S7. The experimental data for the benzene + MeOH, Oct + DCE, and PrDiol + DMF solutions were obtained from previous studies.^7,11^

The solvatochromism of iQ in water + MeOH and in water + EtOH has different origins, as revealed by the Kamlet–Abboud–Taft linear relationship. The α, β, and π* solvents parameters for these solvent mixtures were determined by Buhvestov,^41^ and Figure S2 shows that the hypsochromic shift is given by the HBD property of the binary mixture only in water + MeOH. Polarizability and HBA caused the bathochromic shift in both solutions, but the effect was stronger in water + EtOH.

The dependence of the *E*_T_ values on the mole fraction of the protic solvent, *x*_2_, took different shapes, as expected (Figure 5). The plots in Figure 5a show the influence of the alcohol’s hydrophobic chain in the aqueous binary mixtures of iQ: the experimental data points of water + MeOH are very close to the ideal mixture with a very small positive deviation, while those of water + EtOH are negatively departed from ideality. For the MeOH–water solution, the linearity can be explained by the similarity between the two solvents, as perceived by the iQ molecule. The type and intensity of intermolecular H-bonding of iQ with water or methanol seem to be almost equal. Instead, in the EtOH –water mixture, iQ preferred to be surrounded by water molecules across the entire composition range (negative Δ*E*_T_ values, Δ*E*_T_ = *E*_T,exp_ *– E*_T,calc_). We believe the reason is EtOH’s hydrophobicity and the dispersive forces among the alkyl tails.^42^ The solvation isotherms of benzene + MeOH (Figure 5b) and DMF + PrDiol (Figure 5c) solutions are sigmoidal, with the inflection point located at *x*_2_ = 0.2. The plots indicate a three-step, four-state solvation process and the presence of dispersive interactions at the extremes of the solvent mole fraction. The hyperbolic isotherm of DMSO and DMF with MeOH (Figure 5b), and of DCE with Oct (Figure 5c), is analogous to a simple 1:1 binding process. Similarly to water + EtOH (Figure 5a), the Diox + EG mixture has a concave isotherm (Figure 5c), so the negative Δ*E*_T_ values point to the domination of a constant number of dioxane molecules in the first solvation shell over the whole composition range.

**Figure 5:**
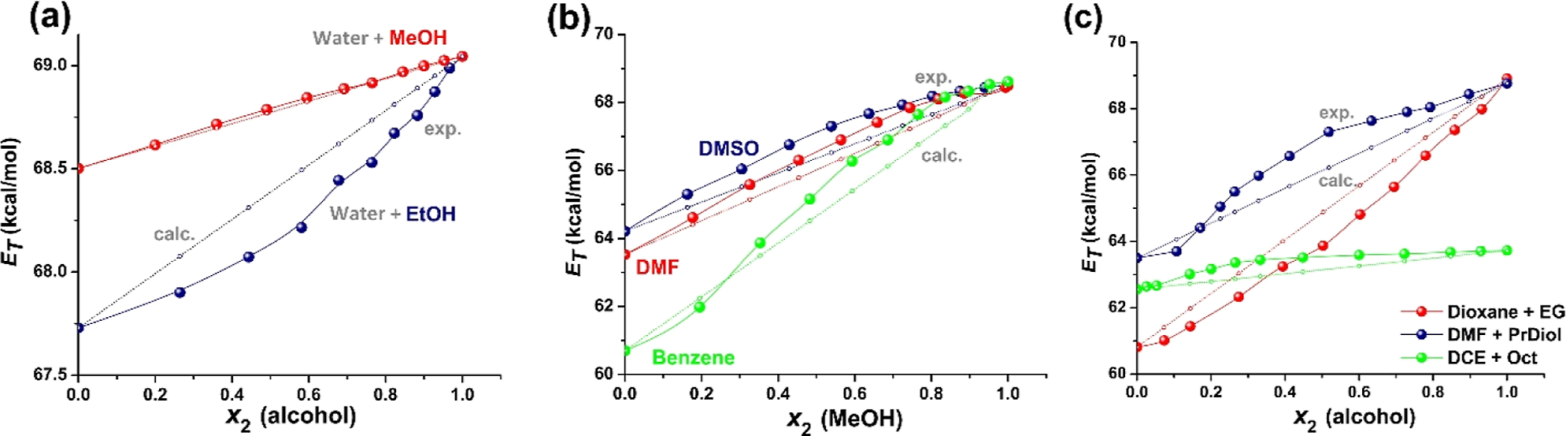
Polarity *E*_T_ parameter of iQ as a function of the mole fraction of the polar cosolvent, *x*_2_, for the mixtures (a) water + MeOH (red)/EtOH (blue); (b) benzene/DMF/DMSO + MeOH; and (c) Diox + EG, DMF + PrDiol and DCE + Oct.

Generally, the preferential solvation parameter δ_s,2_ of iQ by the protic cosolvent S2 can be defined by the expression given by Chatterjee and Bagchi:^43^

$$\delta_{s,2}=x_{2}^{s}-x_{2}$$

where *x*_2_^s^ is the mole fraction of S2 in the first solvation shell of iQ and *x*_2_ is the mole fraction of S2 in the initial bulk mixture. For δ_s,2_ > 0, cosolvent S2 preferentially solvates iQ; for δ_s,2_ < 0, iQ is solvated by the base solvent S1. The values of *x*_2_^s^ are obtained from the spectral data in the individual solvents, according to eqs 3 and 4 in the simplest approach:^43^

3 $$x_{2}^{s}=\frac{E_{T}-E_{T1}}{E_{T2}-E_{T1}}$$

4 $$E_{T}=E_{T1}{\cdot x}_{1}^{s}+E_{T2}{\cdot x}_{2}^{s}$$

In the first place, plotting *x*_2_^s^ as a function of the mole fraction of S2 in the bulk mixture (Figure 6) yields curves analogous to *E*_T_ versus *x*_2_ from Figure 5. The largest preferential solvation is observed in those mixtures that are not based on two protic solvents, as expected (Figure 6a compared to Figure 6b). Thus, the DCE + Oct solution has the highest δ_s,2_ value, reaching 0.4 kcal/mol at *x*_2_ = 0.3 (Figure 6c). In contrast, the preference for the base solvent S1 is observed in the water + EtOH and Diox + EG solutions, with negative δ_s,2_ values and a minimum in δ_s,2_ at *x*_2_ = 0.5. The two-step solvation mechanism is valid for DMF + PrDiol, benzene + MeOH, and, surprisingly, water + MeOH as δ_s,2_ < 0 kcal/mol for *x*_2_ ≤ 0.2 and δ_s,2.max_ occurs at *x*_2_ = 0.5. The biphasic character of the water + MeOH mixture was not apparent when only the *E*_T_ values were analyzed (Figure 5a). The values of the excess energy in hydroalcoholic solutions are 0.03 kcal/mol for water + MeOH and −0.214 kcal/mol for water + EtOH. These are much lower than 0.063 eV (1.45 kcal/mol) reported by Tanaka et al. for Brooker’s merocyanine in water + MeOH^44^ or 2 kcal/mol for a ferrocyanide­(II) complex in water + MeOH in the work of Papadakis.^15^ The main reason is the double H-bonding acceptance of iQ through the carbonyl sites, allowing it to form intermolecular bridges with the solvent on both carboethoxy chains. In contrast, Brooker’s merocyanine (BM) possesses one carbonyl group, while the ferrocyanide­(II) complex contains nitrile groups, which are weaker bases than the ester carbonyl. In addition, both BM and the ferrocyanide­(II) complex are more hydrophobic than iQ, which leads to the manifestation of dispersive forces. The organization of the coordinating solvent around BM or the ferrocyanide­(II) complex must be stronger than around iQ.

**Figure 6:**
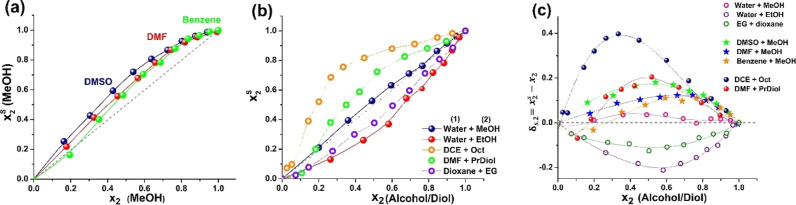
Composition-dependent mole fraction of the cosolvent *x*_2_^s^ in the first solvation shell (a, b) and the preferential solvation parameter δ_s,2_ of iQ (c).

Within the preferential solvation approach of Skwierczynski and Connors,^44^ with *m* solvent molecules in the microsolvation sphere, the mixture’s composition around iQ changes according to two processes:

5a $${\mathrm{iQ}}_{i}{\left(S1\right)}_{m}+mS2⇄{\mathrm{iQ}}_{i}{\left(S2\right)}_{m}+mS1$$

5b $${\mathrm{iQ}}_{i}{\left(S1\right)}_{m}+\frac{m}{2}S2⇄{\mathrm{iQ}}_{i}{\left(S12\right)}_{m}+\frac{m}{2}S1$$

where the iQ molecules can be solvated by solvent S1 (iQ_*i*_(S1) and mole fraction *x*_1_^s^), by S2 (iQ_*i*_(S2) and mole fraction *x*_2_^s^), or by the one-to-one intermolecular complex from S1 and S2 (iQ_*i*_(S12) and mole fraction *x*_12_^s^, eq 5b). The last equation is important for interactions among solvents and for synergistic solvation phenomena.^17,18^ The additivity law (eq 6) must be obeyed:

6 $$x_{1}+x_{2}=x_{1}^{s}+x_{2}^{s}+x_{12}^{s}=1$$

The proportionality relationship between the mole fractions of solvents S1 and S2 in the bulk and in the first solvation shell is given by the preferential solvation parameters *f*_2/1_ and *f*_12/1_, as defined by Bosch and Rosés:^17,18^

7a $$f_{2/1}=\frac{x_{2}^{s}/x_{1}^{s}}{{\left(x_{2}/x_{1}\right)}^{m}}$$

7b $$f_{12/1}=\frac{x_{12}^{s}/x_{1}^{s}}{\sqrt{{\left(x_{2}/x_{1}\right)}^{m}}}$$

where *f*_2/1_ and *f*_12/1_ quantify the probability of the solute to be solvated by solvent S2 and the intersolvent complex S12, respectively, with reference to the base solvent S1. The ICT transition energy of iQ1 when solvated by the S12 pair of solvents, *E*_T12_, is included in the experimentally observed *E*_T_ values from eq 4, so in the Bosch–Rosés model, it becomes

8 $$E_{T}=E_{T1}{\cdot x}_{1}^{s}+E_{T2}{\cdot x}_{2}^{s}+E_{T12}{\cdot x}_{12}^{s}$$

Within this model, the local mole fractions *x*_1_^s^, *x*_2_^s^, and *x*_12_^s^ are derived from eqs 6, 7a, and 7b and have the following expressions as a function of the mole fraction of the protic cosolvent S2 in the bulk mixture:

9a $$x_{1}^{s}=\frac{{\left({1-x}_{2}\right)}^{m}}{{\left(1-x_{2}\right)}^{m}+f_{2/1}{\left(x_{2}\right)}^{m}+f_{12/1}\sqrt{{\left(x_{2}\cdot \left(1-x_{2}\right)\right)}^{m}}}$$

9b $$x_{2}^{s}=\frac{f_{2/1}{\left(x_{2}\right)}^{m}}{{\left(1-x_{2}\right)}^{m}+f_{2/1}{\left(x_{2}\right)}^{m}+f_{12/1}\sqrt{{\left(x_{2}\cdot \left(1-x_{2}\right)\right)}^{m}}}$$

9c $$x_{12}^{s}=\frac{f_{12/1}\sqrt{{\left(x_{2}\cdot \left(1-x_{2}\right)\right)}^{m}}}{{\left(1-x_{2}\right)}^{m}+f_{2/1}{\left(x_{2}\right)}^{m}+f_{12/1}\sqrt{{\left(x_{2}\cdot \left(1-x_{2}\right)\right)}^{m}}}$$

By combining eqs 8 and 9a–9c, the experimental *E*_T_ values are connected to the preferential solvation parameters *f*_2/1_ and *f*_12/1_ by the general relationship

10 $$E_{T}=\frac{E_{T1}{\left(1-x_{2}\right)}^{m}+E_{T2}\cdot f_{2/1}{\left(x_{2}\right)}^{m}+E_{T12}\cdot f_{12/1}\cdot \sqrt{{\left(x_{2}\cdot \left(1-x_{2}\right)\right)}^{m}}}{{\left(1-x_{2}\right)}^{m}+f_{2/1}{\left(x_{2}\right)}^{m}+f_{12/1}\cdot \sqrt{{\left(x_{2}\cdot \left(1-x_{2}\right)\right)}^{m}}}$$

Usually, the solvation process was best described when *m* was close to 2 [14]. Still, in the 2-model approximation and for hydroalcoholic solutions, the behavior at low concentration of alcohol is completely described when a correction parameter is introduced in the form of Δ*E*_T_ ^18,41^

11a $$E_{T}=\frac{E_{T1}{\left(1-x_{2}\right)}^{2}+E_{T2}\cdot f_{2/1}{\left(x_{2}\right)}^{2}+E_{T12}\cdot f_{12/1}\cdot x_{2}\cdot \left(1-x_{2}\right)}{{\left(1-x_{2}\right)}^{2}+f_{2/1}{\left(x_{2}\right)}^{2}+f_{12/1}\cdot x_{2}\cdot \left(1-x_{2}\right)}+\Delta E_{T}$$

11b $$\Delta E_{T}=\frac{k\cdot {f_{2/1}{\left(x_{2}\right)}^{2}[\left(1-x_{2}\right)}^{2}+f_{12/1}\cdot \left(1-x_{2}\right)\cdot x_{2}/2]}{{\left({1-x_{2})}^{2}+f_{2/1}{\left(x_{2}\right)}^{2}+f_{12/1}\cdot x_{2}\cdot \left(1-x_{2}\right)\right)}^{2}}$$

The correction term Δ*E*_T_ accounts for the structured water at low alcohol concentrations,^17,18^ with k being a proportionality constant. Skwierczynski and Connors^45^ and Bosch and Rosés^17,18^ have proposed a series of simplified equations accounting for nonsynergetic mixtures (*f*_12/1_ → 0 and $E_{T}=\frac{E_{T1}+E_{T2}}{2})$ because not all mixtures exhibit intricate behavior. When applying the simplified models to our experimental data and neglecting the correction parameter Δ*E*_T_, the fitting errors were very large or the nonlinear regression did not converge. We concluded that the two solvents interact with each other in all mixtures, even in the absence of H-bonding groups, and the electrostatic forces play a major role. The best results with small fitting errors were obtained using the general eqs 10 and 11a, which have been subjected to nonlinear regression for determining the *E*_T12_, *f*_2/1_, *f*_12/1_, and *k* parameters. The results are listed in Table 3. The graphical representations of local mole fractions *x*_1_^s^, *x*_2_^s^, and *x*_12_^s^ are shown in Figure S3.

**Table 3:** Values of the Model Parameters for the Binary Mixtures

Binary solvent mixture	eq	*E*_T1_	*E*_T2_	*E*_T12_	*f*_2/1_	*f*_12/1_	*k*	RSS^2^ ^a^	SDD^b^
Water + MeOH	([Disp-formula eq10]), *m* = 2	68.5	69.05	68.91	0.39	1.55		0.999	0.006
Water + EtOH	([Disp-formula eq10]), *m* = 2	67.73	69.05	67.99	0.74	1.69		0.997	0.028
DMSO + MeOH	([Disp-formula eq11a])	64.22	68.5	68.1	0.83	1.69	–0.0006	0.999	0.036
Benzene + MeOH	([Disp-formula eq10]), *m* = 2	60.68	68.7	63.58	2.17	1.41		0.999	0.113
DMF + MeOH	([Disp-formula eq10]), *m* = 2	63.53	68.47	68.33	0.41	1.29		0.999	0.031
DCE + Oct	([Disp-formula eq11a])	62.53	63.72	66.71	0.58	0.91	–5.69	0.997	0.030
DMF + PropDiol	([Disp-formula eq11a])	63.32	68.77	68.13	0.11	3.50	0.0015	0.998	0.112
Diox + EG	([Disp-formula eq10]), *m* = 3	60.84	68.75	64.77	0.29	2.19		0.998	0.138

a RSS = residual sum of squares.

b SDD = standard deviation.

Three peculiarities were noted: (i) applying eq 11a without the correction term Δ*E*_T_, that is, eq 10 with *m* = 2, worked best for the hydroalcoholic solutions of water with MeOH and EtOH and the mixtures of MeOH with benzene and DMF; (b) eq 11a holds for DMSO + MeOH, DCE + Oct, and DMF + PrDiol; and (c) eq 10 with *m* = 3 is only valid for the Diox + EG binary mixture because any other variant resulted in the instability of the fitting parameters.

With regard to the values of the solvation parameters in Table 3, the following remarks could be made.

(i) With the exception of the DCE + Oct mixture, the values of *E*_T12_ are in between *E*_T1_ and *E*_T2_, suggesting the lack of synergistic effects.
(ii) Still, *E*_T12_ of Water + MeOH is almost equal to *E*_T2_; within the error limits, we consider that a MeOH–water complex is formed, according to the H-bonding capacities of both solvents. The synergism of MeOH–water combination is well-known,^14,19,41,42^ but for iQ, this mixture is only slightly synergistic. This fact suggests that the H-bond network formed around iQ by MeOH and water is strongly perturbed by other factors, such as the H-bonds made by the ester carbonyl with the hydroxyl groups of MeOH and water and the dipole moment of iQ.
(iii) All *f*_2/1_ parameters are smaller than unity, with the lowest being those for the sigmoidal DMF + PropDiol mixture. It clearly indicates that iQ prefers protic solvents in the immediate vicinity, a trend that is of less importance in DMF + PropDiol.
(iv) The solution benzene + MeOH is an outlier due to the excessively higher *f*_2/1_, of 2.17, which is the result of the strong preference of iQ for MeOH.
(v) The *k* parameter, quantifying the enhancement of the water structure around the solute induced by alcohol molecules,^41^ could not be determined for the two hydroalcoholic solutions. The general eq 11a has been successfully applied instead by Buhvestov et al. for Reichardt’s betaine dye, as well as to several aniline and nitrobenzene-based indicators.^41^ In this case, methanol produced a barely sizable enhancement of water organization around Reichardt’s betaine dye, followed by ethanol. The water structuring became important when higher and branched alcohols were used, such as 2-methylpropan-2-ol or propan-2-ol in the studies of Rosés et al.^18^ It can be deduced, in agreement with the conclusions of Rosés et al.^18^ and Buhvestov et al.,^41^ that the water organization around a solute at low alcohol concentrations is inversely proportional with the H-bonding donor capacity of the alcohol (α) and clearly depends on its hydrophobicity. The self-association of water molecules is promoted by the hydrophobic part of the alcohol, with the same mechanism observed by Rastrelli et al. in the formation of alcohol clusters in alcohol–acetonitrile binary mixtures.^46^ At the same time, the dispersive interactions among the alcohol chains tend to replace the hydrogen bonding in the mixture with the higher alcohols.^46^ Therefore, the magnitude of dispersive interactions is higher in water + ethanol solutions than in water + methanol solutions.

The variation of the local mole fractions of S1, S2, and S12 in the cybotactic region of the iQ molecule, shown in Figure S3, suggests that solvent–solvent complexes were formed in every solution, to various degrees. The local compositions are nonlinear with respect to *x*_12_^s^, and a sigmoid behavior appears for the local mole fraction of MeOH in the benzene + MeOH mixture (Figure S3c). The microsphere of solvation became rich in the protic solvent, S2, at different mole fractions, *x*_2_, which is 0.6 for DCE + Oct, Diox + EG, or water + MeOH and 0.4 for benzene + MeOH.

A preliminary conclusion is that the iQ molecules intervene in the solvent S1–solvent S2 network, so the results in hydroalcoholic solutions do not align with similar studies in the literature.^15,17,18,42^ This fact is attributed to the hydrogen bonds made at the ester groups of iQ. In the microsolvation region, the water, alcohol, or diol molecules prefer to interact with C=O groups rather than with one another or with the polar cosolvent. The high polarizability of the solvent contributes to this phenomenon through dipole–dipole interactions.

#### 3.3.2 The Statistical Cell Model of Ternary Solutions

This model allows the estimation of the interaction energy between two molecules. The mathematical dependence between the molar concentrations of the two solvents in the whole solution and their average weights in the first solvation shell of the solute molecule^47,48^ is as follows

12 $$\ln\frac{x_{2}^{s}}{{1-x}_{1}^{s}}=\ln\frac{x_{2}}{x_{1}}+\frac{\omega_{1}-\omega_{2}}{kT}$$

where *x*_1_^s^ and *x*_2_^s^ are the average statistic weights of the two types of solvent molecules in the first solvation shell, identical to those of the Bosch–Rosés model; *x*_1_ and *x*_2_ are the mole fractions of the solvents in the whole mixture. Quantities ω_1_ and ω_2_ signify the interaction energy in the (solute–S1) and (solute–S2) molecular pairs. When one of the two liquids exerts a weak interaction on the spectrally active molecule, this difference approximates the interaction energy between the solute and the active solvent molecule.

This model will be applied to alcoholic solutions, namely, benzene/DMF/DMSO + MeOH and water + MeOH/EtOH. The dependence of $\ln\frac{x_{2}^{s}}{1-x_{1}^{s}}$ on $\ln\frac{x_{2}}{x_{1}}$ is illustrated in Figure S4. Linear fitting of the experimental data indicates that the cell model for ternary solutions applies to our solutions. From the intercept, the interaction energies in (iQ–solvent) molecular pairs were estimated, and the results are listed in Table S8. They are the highest in the water + MeOH and water + DMSO mixtures, of 4.1 and 3.2 × 10^21^ J, respectively. The lowest is in water + benzene, of 1.2 × 10^21^ J, highlighting the absence of strong interactions, of H-bonding type, between iQ and benzene, and that only iQ–methanol associations can form.

### 3.4 Explicit Solvation Models of iQ

To analyze the type and strength of intermolecular interactions between iQ and ten solvents, we employed a microscale model with explicit solvation. Two to six explicit solvent molecules were placed around one iQ molecule, especially near the ester groups, and the geometry was optimized using DFT-B3LYP/cc-pVDZ. In the particular case of water/small alcohols/diols, modeling is very complicated because the intermolecular complexes [iQ–water/alcohols] have many degrees of freedom. Therefore, we created different initial geometries, with the solvent molecules having different initial coordinates around iQ. Geometry optimization of such large complexes was performed using the less computationally expensive P3M method. The lowest-energy ensembles were identified, and we used them to calculate the single-point energy (DFT/CAM-B3LYP/aug-cc-pVDZ) and the vertical transition energy (TD-DFT/CAM-B3LYP/aug-cc-pVDZ). All calculations used the implicit SMD solvation model, which accounts for the long-range electrostatic effects of the solvent, and the counterpoise correction to account for the basis set superposition errors (BSSE).

Geometry optimization of the microsolvation model comprising iQ and up to six solvent molecules revealed that the main source of interactions was the ester carbonyl. Large solvent networks composed of small molecules such as water, methanol, or ethanol surround the solute. This behavior was anticipated and is usually analyzed using molecular dynamics with solvent cages.^49,50^ Participation of the C^–^ or N^+^ sites was not particularly observed because carbonyls are strong H-bond acceptors (β_2_^H^ = 0.45 ^16^) and the C^–^ or N^+^ atoms are partially protected by the carboethoxy chains. Alcohols and diols form stable single or double H-bonds with C=O, which was expected, and, occasionally, with the oxygen atom on the chain. Although they are H-bond acceptors, DMF and DMSO did not interact with iQ at potential H-donor sites; instead, they preferred to reside near the ester groups. Regardless of the solvent, the iQ molecule undergoes a series of conformational changes to properly interact with the partner (Figure S5), consisting of rotations around the N^+^–C^–^ bond, enlargement of the main C–C^–^–C angle in the dicarboethoxy fragment, and departure from coplanarity of the two carboethoxy chains. The free movement of the dicarboethoxy arms is allowed more in noncoordinating solvents than in alcohols or water as the bifurcated H-bonding at C=O groups tends to “freeze” the structure in a conformation as planar as possible.

#### 3.4.1 The 1:2 Model Complexes

Given that the most probable acceptor sites are the oxygen atoms of the carbonyl ester groups, we analyzed the formation of complexes between iQ and two solvent molecules. We consider that both carbonyl groups of iQ have equal probability to make one intermolecular H-bond at least and that a complex [iQ–2 solvent molecules] can represent any intermolecular ensemble, including those without any direct H-bond at C=O. The electrostatic potential maps (ESP) drawn in Figure 7 show that the aromatic rings suffer a charge modulation of the isoquinolinium ring, resulting from differences in solvation of H-bond acceptor groups. Representative ESP values were taken as maxima on the surface in several critical points: over the aromatic rings and near the O_21_ oxygen atom.^51^ Most of the negative charge is confined in the double ester moieties, and the polyheterocycle is almost neutral. For the isolated iQ molecule, the electrostatic potential over the benzene ring is around 2.75 kcal/mol, over the *N*-containing ring is around 8 kcal/mol, and at O_21_ is −45 kcal/mol. The ESP values over the benzene ring changed slightly upon solvation in aprotic solvents, but changes with +15 kcal/mol are observed with explicit coordinating solvents (ethylene glycol or water), indicating a strong polarization effect. Explicit solvation with *N*,*N*-dimethylformamide deepened the ESP of carbonyl groups by −0.5 kcal/mol, led to the depletion of the benzene ring from electrons (ESP of +9.08 kcal/mol), and increased the dipole moment from 4.23 to 5.40 D. The last effect is surprisingly strong upon solvation in DMSO as the dipole moment reached 9.43 D, and the ESP of the isoquinolinium ring is very asymmetric. The dipole moment increased for the H-bonded complexes (with water, alcohols, and diols), reaching 6.80 D for [iQ–ethylene glycol] (Figure 7).

**Figure 7:**
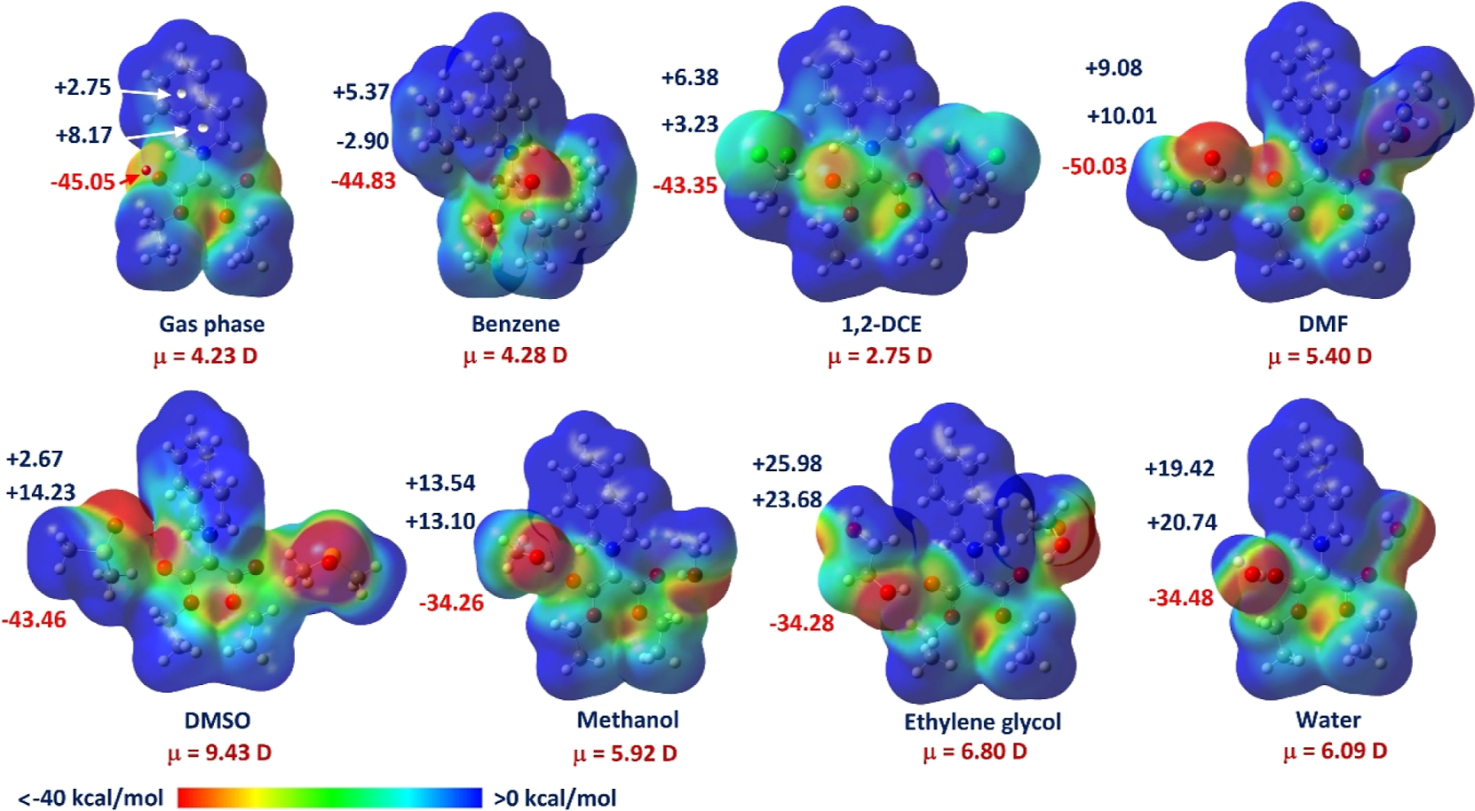
Effect of solvation on the electrostatic potential map of an intermolecular complex [iQ–2 solvent molecules]. Numbers indicate the ESP taken over the two aromatic rings and near the O_21_ oxygen atom, positions indicated by the red and white dots, respectively. Electron density isosurface = 0.001 e/au^3^.

The intermolecular interaction energy between iQ and two solvent molecules, in implicit SMD, was calculated as the difference between the energy of the trimer and the energy of the individual solute and of the solvent. The solvent dependence of the binding energy is plotted in Figure S6 as a function of both polarizability and H-bonding donor ability of the solvent. The interaction energy in the [iQ–benzene] complex is very low, of barely −17.27 kcal/mol, due to the lack of effective specific interactions. Coordinating solvents did not necessarily lead to very stable [solute–solvent] complexes, except for water, but relative stability was obtained in very polar solvents due to polarization and dispersion phenomena. All alcohols and diols are clustered into a zone with a binding energy of −60 to −75 kcal/mol, despite their high α parameter. The polarization effects induced by DMF increased the binding energy to nearly −130 kcal/mol, whereas the [iQ–1,4-dioxane] complex has a binding energy of approximately −94 kcal/mol. These results complete the Kamlet–Abboud–Taft analysis in pure solvents presented in Section 3.3, where the polarizability of the solute was partially responsible for the solvatochromic behavior.

#### 3.4.2 Modeling the 1:*n* Complexes

We investigated the relationship between the number of explicit solvent molecules surrounding an iQ molecule and the calculated UV–vis spectrum to match the experimental data as closely as possible. We chose two solvents with the opposite polarity and H-bonding abilities: benzene and water. Geometry optimization of the models was done at a low level of theory (AM1). Single-point DFT calculations were performed by using the B3LYP functional, the 6-31G split valence basis set, and the SMD solvent model. To analyze the solvatochromic shift in the UV–vis spectra, the vertical transition energies were calculated using (TD)-DFT-CAM-B3LYP/aug-cc-pVTZ and TD-DFT/CAM-B3LYP/6-311G­(d,p) with the dev2-SVP effective core potential.

Microsolvation of iQ in benzene was limited to 4 molecules in its vicinity due to computational resources. Two types of 1:2 complexes were designed, differentiated by the placement of benzene molecules: near the carbonyl groups (system 1) or parallel with the polyheterocycle (system 2). The solvated structures for 1:2 and 1:4 complexes, shown in Figure 8a near the calculated UV spectra, do not contain any intermolecular H-bonds, as expected. The benzene molecules may prefer a parallel orientation relative to the isoquinolinium ring, similar to π–π stacking, whereas there is no particular alignment near the dicarboethoxy chain. The energetics of the 1:2 pairs with benzene near the oxygen atoms is slightly lower than that near the polyheterocycle (+15 kcal/mol as compared with +25 kcal/mol). The energy of the 1:4 pairs is +80 kcal/mol on average.

**Figure 8:**
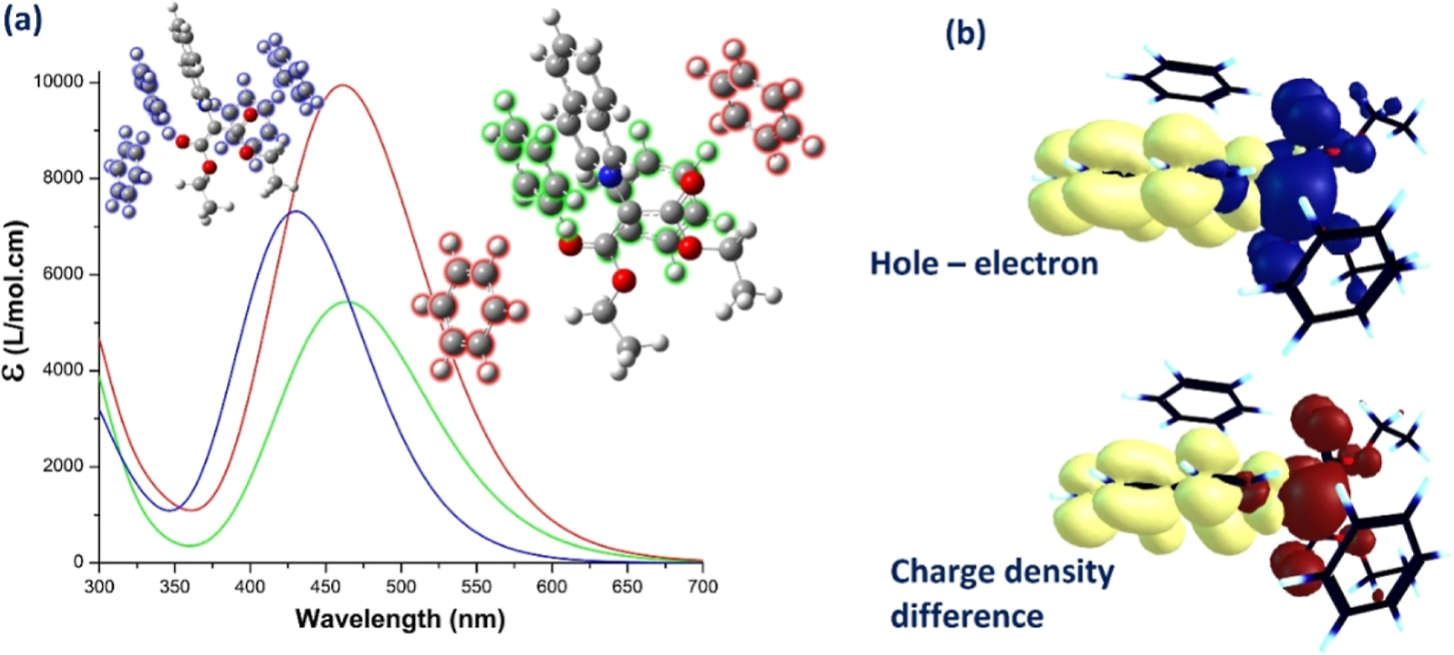
Explicit solvation of iQ in benzene: (a) calculated electronic absorption spectra and the optimized geometry of molecular models; (b) electronic excitation analysis of the ICT band for a 1:2 complex: distribution of the hole–electron pair and the charge density difference. Color code of the lobes: hole—blue, electron—yellow, positive phase—yellow, and negative phase—brown.

Figure 8a depicts the effect of explicit solvation with two and four benzene molecules on the electronic absorption spectrum of iQ. Although there is a small difference of 2 nm in the maximum of the ICT/S_1_ band between the two types of 1:2 systems (λ_max1_ = 461 nm, *f*_1_ = 0.134 and λ_max2_ = 463 nm, *f*_2_ = 0.245), the oscillation strength nearly doubles when the benzene molecules are near the oxygen atoms (system 1, red spectrum in Figure 8a). When four benzene molecules are placed around iQ, the calculated UV–vis spectrum shows a significant 30 nm blue shift of ICT/S_1_ and is 40 nm lower than the experimental spectrum. The hole–electron distribution and the charge density difference between the ground and the first excited state, shown in Figure 8b, look similar to those in implicit solvation.

Calculations with explicit water were based on (i) the size of the iQ molecule, which allows for a large number of water molecules to float around it; (ii) the ability of C=O to simultaneously bind two protons; and (iii) the oxygen atom on the carboethoxy chain being reactive and able to participate in the binding energy. At the same time, the large number of H-bonding opportunities increases the number of binding combinations available for water. Given the computational time required for large water clusters and the available resources, we restricted the calculations to 1:2 to 1:8 model systems in which water molecules were placed near the reactive units of iQ. We used four models to account for different water concentrations in the solutions: with two H-bonds (2 water molecules, each one per C=O group), three H-bonds (three water molecules, with one H-bond on one carbonyl and two H-bonds on the other carbonyl), two double H-bonds (4 water molecules, two H-bonds per C=O), and four H-bonds at C=O and one/two H-bonds at C–O (six to eight water molecules).

Placement of 6–8 waters near iQ, in various positions, led to the formation of small water networks around the molecule and of bifurcated and trifurcated H-bonds at the ester carbonyl (Figure 9a). But, transient H-bonding interactions among water molecules continuously shape H-bonded structures, changing the number and geometry of hydrogen bonds, especially when the number of water molecules exceeds six. The binding energy increased almost linearly with the number of water molecules (Figure 9a) and began to plateau when the number of hydrogen bonds between water and iQ reached a threshold. This was observed if the C–O groups were involved in intermolecular interactions as the number of C–O H-bonds with water did not contribute much to the global binding energy. Instead, the number of H-bonded carbonyl oxygen atoms determined the binding energy, therefore the energetics of the complex, and subsequently the maximum and the intensity of the ICT/S_1_ band (Figure 9b). The 1:2 complex with one H-bond per C=O group has a calculated S_1_ state at λ_max_ ≈ 450 nm, with *f* = 0.155 (green spectrum in Figure 9c). Solvation with four water molecules near the carbonyl groups, giving rise to two bifurcated H-bonds, produces a hypsochromic shift (Δλ = −40 nm) accompanied by a hyperchromic effect (*f* = 0.233), exemplified by the dark yellow spectrum in Figure 9b.

**Figure 9:**
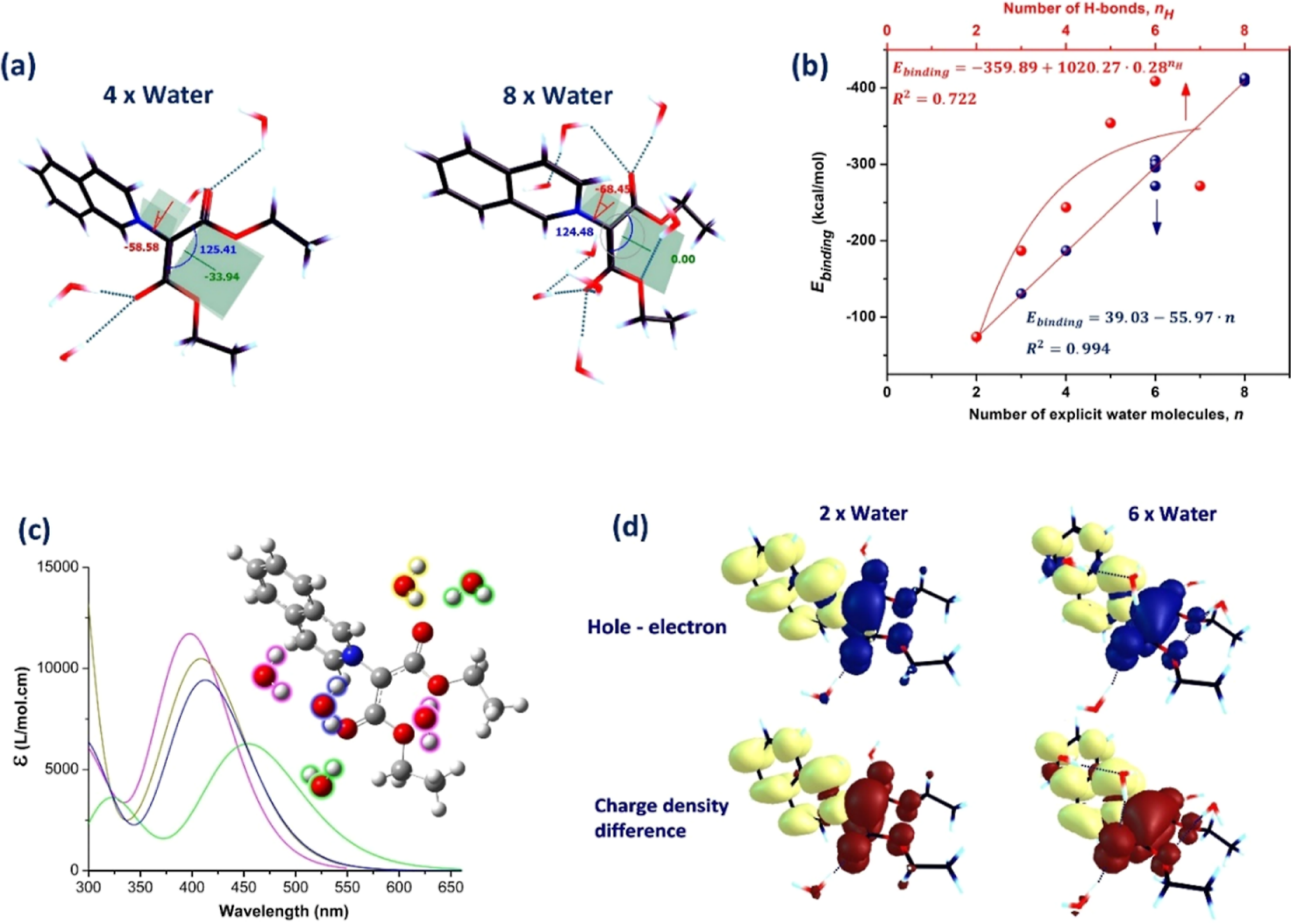
Explicit solvation of iQ with water molecules in the SMD model: (a) optimized geometry of representative 1:4 and 1:8 complexes; (b) *E*_binding_ as a function of neighboring water molecules and number of H bonds, and the fit as the red line; (c) calculated UV–vis spectra of solvated iQ with *n* water molecules: *n* = 2 (green), *n* = 3 (yellow), *n* = 4 (blue), and *n* = 6 (magenta); and (d) electronic excitation analysis for 1:2 and 1:6 model complexes. Color code of the lobes: hole—blue, electron—yellow, positive phase—yellow, and negative phase—brown.

The calculated UV–vis spectrum for three to five explicit water molecules is reasonably close to the experimental data, giving λ_max_ ≈ 410 nm (*f* = 0.254), as compared with the experimental λ_exp_ = 414 nm. The calculations for 1:6 and 1:8 explicit waters yielded λ_max_ = 398 nm and λ_max_ = 394 nm, respectively, with a deviation of Δλ = −16 to −20 nm relative to the experimental results. The analysis of the electronic excitation, shown in Figure 9d, reveals that the hole and electron distributions partially overlap when the number of surrounding water molecules is high. This conclusion confirms the analysis in implicit solvent, where the mixed character of the S_0_ → S_1_ transition (ICT + LE) was observed in strongly coordinating solvents.

We may observe that calculated UV–vis spectra in explicit solvation are very sensitive to the placement of the solvent molecule around iQ. This is because the electrostatic dipole–dipole interactions affect the energetics of the solute and modify the magnitude of the charge transfer. The effect is stronger in polar protic solvents as the calculated maximum of the ICT/S_1_ band shifts by almost 15 nm when the number of explicit water molecules goes from four to eight. These simulations with varying numbers of solvent molecules may account for the behavior of iQ in binary mixtures of alcohols and water with an aprotic solvent. According to experimental data in pure solvents, the position of the maximum of the ICT band differs by only 3 nm between methanol and water. We recall that in benzene + MeOH, λ_max_ blue-shifts from 470 to 417 nm, while in DMSO + MeOH, λ_max_ goes from 450 to 417 nm. In a broad sense, we may translate explicit solvation with water to that with MeOH, with the amendment that the MeOH network is not so strong. The above results with explicit solvation describe the experimental data in alcohol mixtures reasonably well.

## 4 Conclusions

Iso-quinolinium dicarboethoxy methylid (iQ), a zwitterionic compound with the positive and negative charges next to each other, is a very sensitive molecule to the hydrogen bonding and polarizability properties of the solvent. Its negative solvatochromism has been thoroughly analyzed using theoretical models combined with quantum–chemical calculations, the latter using a hybrid approach. The composition of the microsphere of solvation, determined by means of the preferential solvation model of Bosch and Rosés, is composed mainly of those solvents with high HB donor properties. The hydroalcoholic solutions of ethanol and methanol in water show no substantial synergism, which does not align with results reported in the literature obtained with other zwitterionic compounds. The difference arises from the presence of ester groups in the iQ structure, which form strong hydrogen bonds with alcohols and water, thereby disrupting the solvent–solvent network around iQ. DFT calculations with and without implicit solvation (SMD model) revealed that iQ behaves like a molecular rotor, and free rotation about the central N^+^–C^–^ bond yields two stable conformers, which are almost indistinguishable in terms of energy. The calculated excitation energies of iQ were reproduced reasonably well in pure solvents. Calculations with explicit benzene or water solvent molecules in implicit SMD solvation provided a hint about the spectral changes when the surroundings of iQ became progressively filled with a strongly coordinating solvent.

## Supplementary Material



## Notes

Tables with the experimental UV–vis data for the binary solvent mixtures and additional figures and tables (PDF)
