# Supplementary material for: Use of a Zwitterionic Isoquinolinium Dye as a Solvatochromic Indicator: Structure of the Microsphere of Solvation and Explicit Solvation Modeling
Source: J Phys Chem B. 2026 May 14;130(21):5355–69. doi: 10.1021/acs.jpcb.6c01103 (PMC13224189; doi:10.1021/acs.jpcb.6c01103)
Supplement: Supplementary file 1 [file jp6c01103_si_001.pdf]

Supporting Information

Use of a Zwitterionic Isoquinolinium Dye as a Solvatochromic Indicator: Structure of the Microsphere of Solvation and Explicit Solvation Modeling

Ovidiu Gabriel Avadanei,[1] Dana Ortansa Dorohoi,[1] Mihaela Iuliana Avadanei[2*]

[1]"Alexandru Ioan Cuza" University of Iasi, Faculty of Physics, 11 Carol I Blvd., 700506, Iasi, ROMANIA

[2]"Petru Poni" Institute of Macromolecular Chemistry, 41A Gr. Ghica Voda,700487, Iasi, ROMANIA

Table S1. Solvents' polarity parameters [1].

No	Solvent	ε_r	n	α	β	π*
**1**	Benzene	2.27	1.5011	0	0.1	0.59
**2**	1,4-Dioxane	2.2	1.4224	0	0.37	0.55
**3**	1,2-Dichloroethane	10.3	1.3729	0	0.1	0.81
**4**	1-Octanol	10.3	1.429	0.77	0.81	0.4
**5**	Ethanol	24.55	1.361	0.98	0.83	0.51
**6**	Methanol	32.62	1.3314	0.98	0.66	0.6
**7**	Propane-1,3- diol	34.69	1.4398	0.76	0.84	0.48
**8**	DMF	36.71	1.4305	0	0.69	0.88
**9**	Ethylene glycol	37.7	1.4318	0.9	0.52	0.92
**10**	DMSO	46.45	1.479	0	0.76	1
**11**	Water	80	1.4459	0.2	0.1	0.69

DMF = N,N-dimethylformamide; DMSO = dimethylsulfoxide.

[1] Y. Marcus, The properties of organic liquids that are relevant to their use as solvating solvents, Chem. Soc. Rev. 22 (1993) 409–416. https://doi.org/10.1039/CS9932200409.

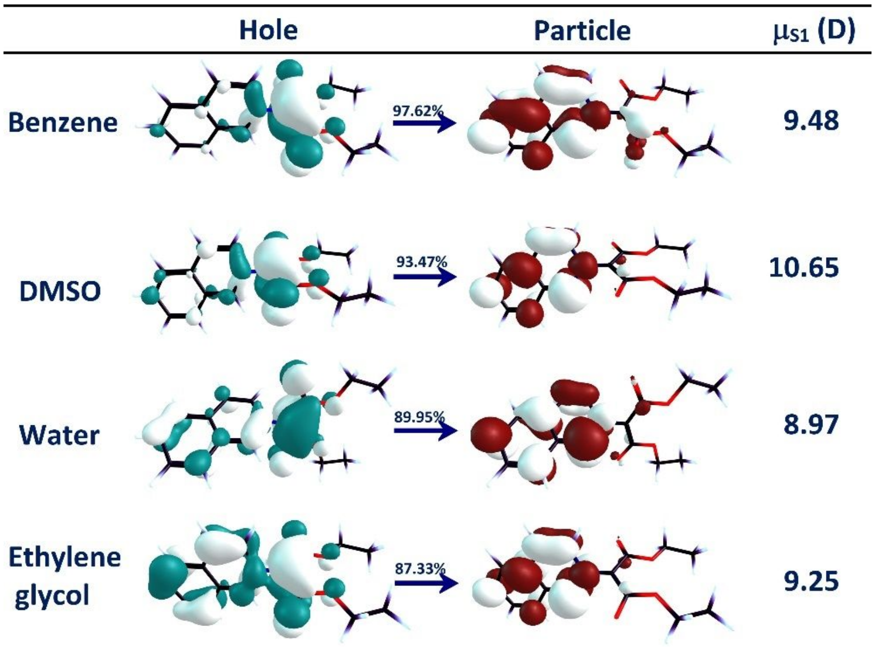

Figure S1. The natural transition orbital pairs of the first excited state for iQ in four representative solvents and the dipole moment, μ_{S1}, in Debye. Color code of NTOs phases: positive phase – white, negative phase – green/brown. Isovalue: 0.0250.

Table S2. Maximum of the ICT band of iQ in the individual solvents, expressed in wavenumbers, wavelength and molar transition energy, E_T.

No	Solvent	Wavenumber, cm^{-1}	Wavelength, nm	E_T, kcal/mol
**1**	Benzene	21230	471	60.696
**2**	1,4-Dioxane	21270	470	60.810
**3**	1,2-Dichloroethane	21880	457	62.554
**4**	1-Octanol	22290	448	63.727
**5**	Ethanol	23690	422	67.729
**6**	Methanol	23960	417	68.501
**7**	Propane-1,3- diol	24050	416	68.758
**8**	DMF	22220	450	63.527
**9**	Ethylene glycol	24104	415	68.913
**10**	DMSO	22460	445	64.213
**11**	Water	24150	414	69.044

DMF = N,N-dimethylformamide; DMSO = dimethylsulfoxide.

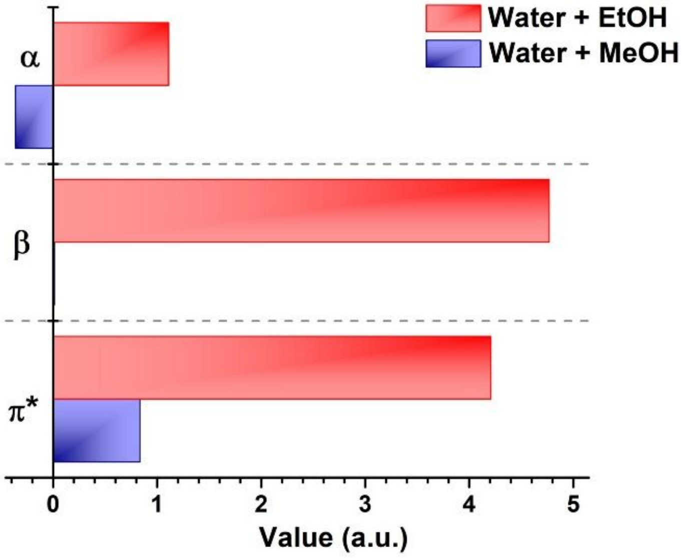

Figure S2. Kamlet-Abboud-Taft linear relationship applied to iQ in binary mixtures water + MeOH and in water + EtOH: α is the H-bond donor parameter, β is the H-bond acceptor parameter, and π* is polarizability.

Table S3. IQ in the binary solvent Methanol (1) + Benzene (2): volumetric (C_1, %), molecular concentration (x_1, %) of methanol; average statistic weight of methanol in the first solvation shell of iQ (p_1%); wavenumber of the ICT absorption band (ν cm^{-1}); and dielectric permittivity of binary solvent, ε.

$x_1\%$	x_1	$ln\frac{x_1}{1-x_1}$	$\nu_t\,(cm^{-1})$	$p_1\%$	$ln\frac{p_1}{1-p_1}$	$p_1 - x_1$	ε
0	0	-	21230	0	-	0	
10	0.195	- 0.142	21680	16.30	-1.64	- 0.032	
20	0.353	- 0.606	22340	40.10	-0.401	0.048	
30	0.483	- 0.068	22790	56.30	0.253	0.080	
40	0.593	0.376	23180	70.40	0.866	0.111	
50	0.686	0.781	23400	78.30	1.283	0.097	
60	0.766	1.186	23660	87.70	1.851	0.111	
70	0.836	1.629	23840	94.20	2.788	0.045	
80	0.897	2.164	23900	96.40	3.288	0.067	
90	0.952	2.987	23970	98.90	4.499	0.037	
100	0	-	24000	100	-		

Table S4. IQ in binary solvent MeOH (1) + DMF (2): volumetric (C_1, %), molecular concentration (x_1, %) of methanol; average statistic weight of MeOH in the first solvation shell of iQ (p_1, %); wavenumber of the ICT absorption band (ν_t, cm^{-1}) and electric permittivity of binary solvent, ε.

$C_1\%$	$x_1\%$	$ln\frac{x_1}{1-x_1}$	$\nu_t\,(cm^{-1})$	$p_1\%$	$ln\frac{p_1}{1-p_1}$	ε
0	0	-	22220	0	-	37.6
10	17.7	-1.537	22600	21.84	-1.28	37.2
20	32.6	-0.726	22940	41.38	-0.35	36.7
30	45.4	-0.185	23190	55.75	0.23	36.3
40	56.4	0.257	23400	67.82	0.75	35.6
50	65.9	0.659	23580	78.16	1.28	35.0
60	74.4	1.067	23730	86.78	1.88	33.5
70	81.9	1.510	23820	91.95	2.44	32.8
80	88.6	2.051	23880	95.40	3.03	32.3
90	99.4	5.110	23940	98.85	4.45	31.7
100	100	-	23960	100	-	31.0

Table S5. IQ in the binary solvent MeOH (1) + DMSO (2): volumetric (C_1, %), molecular concentration (x_1, %) of methanol; average statistic weight of MeOH in the first solvation shell of iQ (p_1, %); wavenumber of the ICT absorption band (ν_t, cm^{-1}) and electric permittivity of binary solvent, ε.

$C_1\%$	$x_1\%$	$ln\frac{x_1}{1-x_1}$	$\nu_t\,(cm^{-1})$	$p_1\%$	$ln\frac{p_1}{1-p_1}$	ε
0	0	-	22460	0	-	54.0
10	16.3	-1.636	22840	25.33	-1.08	52.0
20	30.5	-0.824	23100	42.67	-0.3	50.4
30	42.9	-0.286	23350	59.33	0.38	48.2
40	53.9	0.156	23540	72.00	0.94	46.0
50	63.7	0.562	23670	80.67	1.43	44.0
60	72.5	0.969	23760	86.67	1.87	41.5
70	80.3	1.405	23850	92.67	2.54	38.8
80	87.5	1.946	23900	96.00	3.18	36.0
90	94.0	2.752	23940	98.67	4.3	33.6
100	100	-	23960	100	-	31.0

Table S6. IQ in the binary solvent Water (1) + MeOH (2): molecular concentration (x_1, %) of water; average statistic weight of water in the first solvation shell of iQ (p_1, %); wavenumber of the ICT absorption band (ν_t, cm^{-1}); Kamlet Abboud Taft coefficients (π^*,β, α) electric permittivity of binary solvent, ε.

$x_1\%$	$ln\frac{x_1}{1-x_1}$	$\nu_t\,(cm^{-1})$	$p_1\%$	$ln\frac{p_1}{1-p_1}$	π^*	β	α	ε	
0	-	23960	0	-	0.58	0.74	1.14		0
10	-2.20	24050	47.37	-0.11	0.64	0.74	1.12		37.37
20	-1.39	24035	39.47	-0.43	0.60	0.74	1.09		19.47
30	-0.85	24060	52.63	0.11	0.76	0.74	1.06		22.63
40	-0.41	24080	63.16	0.54	0.82	0.72	1.04		23.16
50	0.00	24095	71.05	0.9	0.88	0.70	1.03		21.05
60	0.41	24105	76.32	1.17	0.95	0.66	1.01		16.32
70	0.85	24124	86.32	1.84	1.01	0.63	1.01		16.32
80	1.39	24134	91.58	2.39	1.06	0.59	1.06		11.58
90	2.20	24143	96.32	3.26	1.11	0.54	1.13		6.32
100	-	24150	100		1.14	0.49	1.23		0

Table S7. IQ in the binary solvent Water (1) + Ethanol (2): molecular concentration (x_1, %) of water; average statistic weight of water in the first solvation shell of iQ (p_1, %); wavenumber of the ICT absorption band (ν_t, cm^{-1}); Kamlet Abboud Taft coefficients (π^*,β, α) and electric permittivity of binary solvent, ε.

$x_1\%$	$ln\frac{x_1}{1-x_1}$	$\nu_t\,(cm^{-1})$	$p_1\%$	$ln\frac{p_1}{1-p_1}$	π^*	β	α	ε	P_1-x1
0	-	23690	0	-	0.51	0.83	0.98		
10	-2.20	23750	13.04	-1.9	0.57	0.84	0.96		3.04
20	-1.39	23810	26.09	-1.04	0.63	0.83	0.93		6.09
30	-0.85	23860	36.96	-0.53	0.68	0.82	0.92		6.96
40	-0.41	23940	54.35	0.17	0.73	0.80	0.91		14.35
50	0.00	23970	60.87	0.44	0.77	0.79	0.90		10.87
60	0.41	24020	71.74	0.93	0.82	0.77	0.89		11.74
70	0.85	24050	78.26	1.28	0.90	0.74	0.88		8.26
80	1.39	24090	86.96	1.9	1.00	0.67	0.87		6.96
90	2.20	24130	95.65	3.09	1.11	0.59	0.97		5.65
100	-	24150	100	-	1.13	0.50	1.26		0

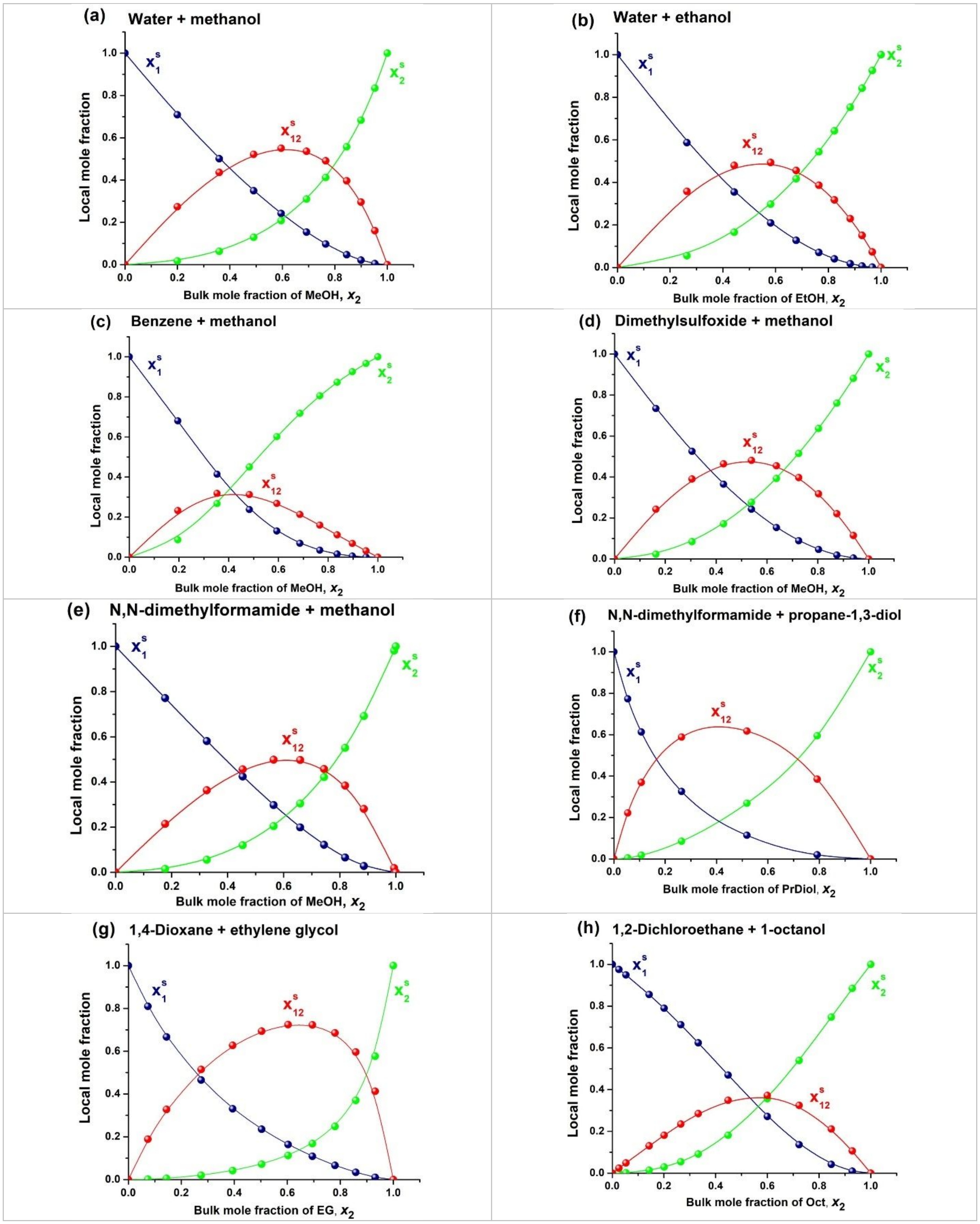
(a) Water + methanol
Local mole fraction
Bulk mole fraction of MeOH, x_2
x_1^s
x_2^s
x_{12}^s
(b) Water + ethanol
Local mole fraction
Bulk mole fraction of EtOH, x_2
x_1^s
x_2^s
x_{12}^s
(c) Benzene + methanol
Local mole fraction
Bulk mole fraction of MeOH, x_2
x_1^s
x_2^s
x_{12}^s
(d) Dimethylsulfoxide + methanol
Local mole fraction
Bulk mole fraction of MeOH, x_2
x_1^s
x_2^s
x_{12}^s
(e) N,N-dimethylformamide + methanol
Local mole fraction
Bulk mole fraction of MeOH, x_2
x_1^s
x_2^s
x_{12}^s
(f) N,N-dimethylformamide + propane-1,3-diol
Local mole fraction
Bulk mole fraction of PrDiol, x_2
x_1^s
x_2^s
x_{12}^s
(g) 1,4-Dioxane + ethylene glycol
Local mole fraction
Bulk mole fraction of EG, x_2
x_1^s
x_2^s
x_{12}^s
(h) 1,2-Dichloroethane + 1-octanol
Local mole fraction
Bulk mole fraction of Oct, x_2
x_1^s
x_2^s
x_{12}^s

Figure S3. Local mole fractions of solvent S1, x_1^s , of solvent S2, x_2^s and of the complex molecule S1 – S2, x_{12}^s around the iQ molecule.

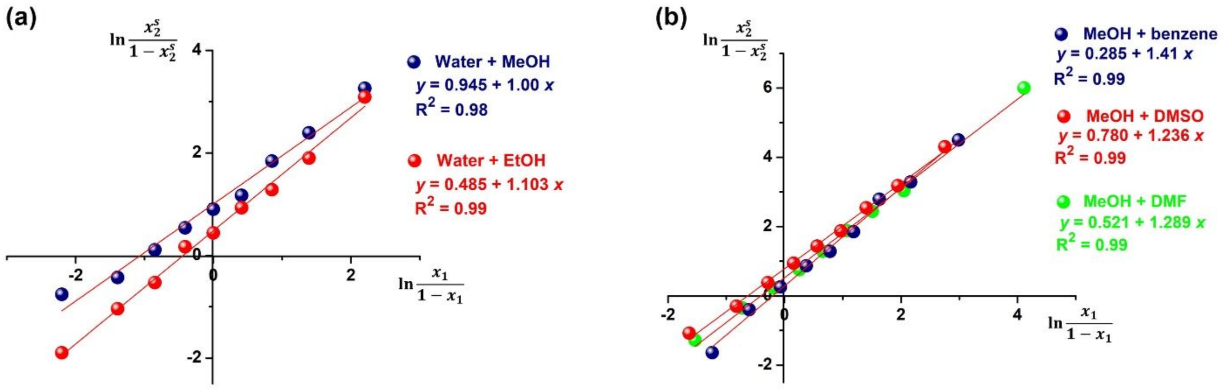

Figure S4. Plots of $ln\frac{x_2^s}{1-x_1^s}$ vs. $ln\frac{x_2}{x_1}$ for iQ in binary solution of: (a) water + MeOH/ EtOH; (b) benzene/DMF/DMSO + MeOH.

Table S8. The interaction energies in the pairs between iQDCM and binary solvents

Binary mixture	Intercept	w_2-w_1, × 10^{21} J
**Water + MeOH**	1.002	4.119
**Water + EtOH**	0.482	1.981
**MeOH + Benzene**	0.285	1.173
**MeOH + DMSO**	0.780	3.206
**MeOH + DMF**	0.521	2.141

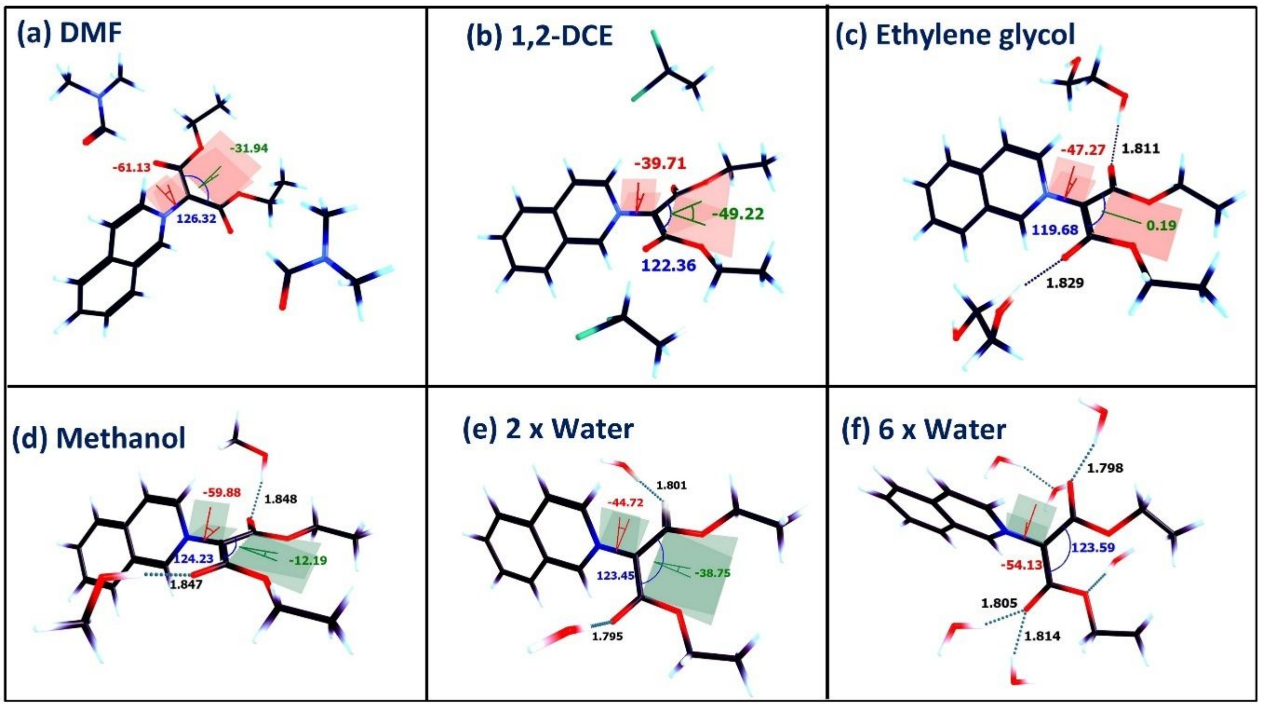

Figure S5. Geometry optimization of 1:2 complexes of iQ with: (a) DMF; (b) 1,2-dichloroethane; (c) ethylene glycol; (d) methanol: (e) two water molecules; (f) four water molecules.

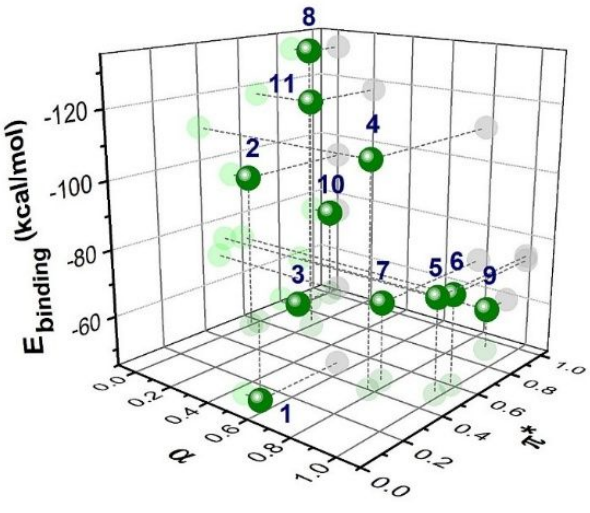

	Solvent	E binding, kcal/mol
1	Benzene	-17.27
2	1,4-Dioxane	-93.26
3	1,2-Dichloroethane	-45.55
4	1-Octanol	-110.03
5	Ethanol	-73.10
6	Methanol	-71.76
7	Propane-1,3-diol	-68.23
8	DMF	-129.53
9	Ethylene glycol	-57.74
10	DMSO	-73.75
11	Water	-116.95

Figure S6. Relationship between $E_{binding}$ of 1:2 complexes and the solvent parameters α (H-bond donor ability) and π* (polarizability).
